# Functionalized ZnO-Based Nanocomposites for Diverse Biological Applications: Current Trends and Future Perspectives

**DOI:** 10.3390/nano14050397

**Published:** 2024-02-21

**Authors:** Ioanna-Aglaia Vagena, Maria-Anna Gatou, Giorgos Theocharous, Pavlos Pantelis, Maria Gazouli, Natassa Pippa, Vassilis G. Gorgoulis, Evangelia A. Pavlatou, Nefeli Lagopati

**Affiliations:** 1 Laboratory of Biology, Department of Basic Medical Sciences, Medical School, National Kapodistrian University of Athens (NKUA), 11527 Athens, Greece; iliannavgn@med.uoa.gr (I.-A.V.); mgazouli@med.uoa.gr (M.G.); 2 Laboratory of General Chemistry, School of Chemical Engineering, National Technical University of Athens, Zografou Campus, 15772 Athens, Greece; mgatou2@mail.ntua.gr (M.-A.G.); pavlatou@chemeng.ntua.gr (E.A.P.); 3 Molecular Carcinogenesis Group, Department of Histology and Embryology, Medical School, National Kapodistrian University of Athens (NKUA), 11527 Athens, Greece; gtheoc@med.uoa.gr (G.T.); pavlospp@biol.uoa.gr (P.P.);; 4 School of Science and Technology, Hellenic Open University, 26335 Patra, Greece; 5 Section of Pharmaceutical Technology, Department of Pharmacy, School of Health Sciences, National Kapodistrian University of Athens (NKUA), 15771 Athens, Greece; natpippa@pharm.uoa.gr; 6 Biomedical Research Foundation, Academy of Athens, 11527 Athens, Greece; 7 Ninewells Hospital and Medical School, University of Dundee, Dundee DD19SY, UK; 8 Faculty Institute for Cancer Sciences, Manchester Academic Health Sciences Centre, University of Manchester, Manchester M20 4GJ, UK; 9 Faculty of Health and Medical Sciences, University of Surrey, Guildford GU2 7YH, UK

**Keywords:** ZnO nanostructures, biomedical applications, synthetic approaches, functionalization, biosensors, immunotherapy, tissue engineering, bioimaging, wound healing, toxicity mechanism

## Abstract

The wide array of structures and characteristics found in ZnO-based nanostructures offers them a versatile range of uses. Over the past decade, significant attention has been drawn to the possible applications of these materials in the biomedical field, owing to their distinctive electronic, optical, catalytic, and antimicrobial attributes, alongside their exceptional biocompatibility and surface chemistry. With environmental degradation and an aging population contributing to escalating healthcare needs and costs, particularly in developing nations, there’s a growing demand for more effective and affordable biomedical devices with innovative functionalities. This review delves into particular essential facets of different synthetic approaches (chemical and green) that contribute to the production of effective multifunctional nano-ZnO particles for biomedical applications. Outlining the conjugation of ZnO nanoparticles highlights the enhancement of biomedical capacity while lowering toxicity. Additionally, recent progress in the study of ZnO-based nano-biomaterials tailored for biomedical purposes is explored, including biosensing, bioimaging, tissue regeneration, drug delivery, as well as vaccines and immunotherapy. The final section focuses on nano-ZnO particles’ toxicity mechanism with special emphasis to their neurotoxic potential, as well as the primary toxicity pathways, providing an overall review of the up-to-date development and future perspectives of nano-ZnO particles in the biomedicine field.

## 1. Introduction

In recent years, nanotechnology has ushered in significant advancements in modern scientific innovation. This field focuses on materials at the nanoscale (1–100 nm). These nano-scaled materials possess distinct attributes, attributable to their minute dimensions, extensive specific surface area, structure, as well as chemical composition, holding promise for a plethora of biomedical applications, including diagnostics, bioimaging, and drug delivery [1,2,3,4,5]. Metal nanoparticles, in particular, stand out for their diverse utility in biomedical, agricultural, optical, and electrical domains, given their exceptional characteristics. A wide array of metals is employed in synthesizing different types of metal nanoparticles, such as FeO, ZnO, TiO_2_, In_2_O_3_, SnO_2_, SiO_2_, Au, Ag, CeO_2_ and CuO nanoparticles [6]. Among these, zinc oxide (ZnO) nanoparticles have garnered considerable attention, because of their distinct attributes.

Zinc oxide nanoparticles represent a highly versatile material, possessing distinctive physico-chemical attributes including notable chemical robustness, an enhanced electrochemical coupling coefficient, a wide spectrum of irradiation absorbance, as well as excellent light stability. The crystalline form of ZnO can manifest in three crystal structures: rock salt, wurtzite, and sphalerite (cubic). Among these, the wurtzite structure is the most thermodynamically stable and commonly occurring in nature, while rock salt structure is rare and typically formed under high pressure. Sphalerite, on the other hand, remains stable under ambient pressure and temperature conditions. Notably, nano-ZnO particles exhibit a diverse range of structural varieties among known nanomaterials, including zero-dimensional (nanospheres and quantum dots) [7,8], one-dimensional (nanorods, needles, helices, springs, fibers, tubes, and wires) [9,10,11,12,13], two-dimensional (nanoplates, nanosheets, nanoribbons, and nanorings) [14,15,16,17], and three-dimensional (flowers, dandelions, snowflakes, coniferous sea urchins, etc.) structures [18,19,20]. Recognized as a prudent and multifunctional inorganic material, zinc oxide is classified as a semiconductor within the II–VI group, situated between the realms of ionic and covalent semiconductor materials. Its broad bandgap (3.37 eV), increased exciton binding energy (60 meV), as well as enhanced thermal and mechanical robustness at ambient temperature render zinc oxide nanoparticles auspicious for various applications. Additionally, owing to its increased specific surface area, reduced toxicity, superior biocompatibility, and biodegradability, zinc oxide holds significance in biomedical and biophilic systems [21,22,23]. Noteworthy is its low biological toxicity, setting it apart from other nanoparticles [24]. Furthermore, ZnO nanoparticles demonstrate exceptional properties, such as antibacterial, antidiabetic, anticancer, wound healing, bioimaging, and UV blocking, making them a cost-effective and biocompatible option in biomedicine [25,26,27,28,29,30].

Two primary approaches are commonly employed in the synthesis of ZnO nanoparticles: (a) top-down and (b) bottom-up methods. In the top-down approach, larger molecules (bulk material) disintegrate into smaller molecules, which are then transformed into nanoparticles. Mechanisms, such as laser ablation and high-energy ball milling, are representative of top-down synthesis methods. Conversely, the bottom-up approach, often termed the constructive method, stands as the inverse of top-down synthesis. This method encompasses processes like sol–gel, microemulsion and biosynthesis/green synthesis [31,32]. However, chemical processes present numerous adversities, including the requirement for expensive chemicals, specialized infrastructure, high energy consumption, and the potential formation of harmful by-products. Moreover, synthesized ZnO nanoparticles may exhibit increased toxicity [33,34,35]. Consequently, alternative synthesis routes employing biosynthesis/green methods have been proposed. These methods replace harmful chemicals with extracts from several plant parts, like flowers, leaves, fruit, stems, and roots, as well as microorganisms, such as bacteria, viruses, fungi and algae [34]. Plant extracts contain diverse phytochemicals, such as tannins, flavonoids, and iso-flavonoids, which serve as both reducing and stabilizing agents [36]. Also, biomolecules present within microorganisms have a vital impact, acting as miniaturized nano-factories. Biosynthesis utilizing microorganisms offers benefits over chemical and biosynthesis employing plant extracts, due to their ease of production. Nonetheless, these procedures are time-consuming, and identifying suitable microorganisms poses a significant challenge [37].

It is widely acknowledged that ZnO-based devices’ efficacy hinges on the microstructure of nano-ZnO structures. Consequently, parameters such as size, orientation, morphology, aspect ratio, and crystals’ density play pivotal roles in assessing the possible applications of nano-ZnO materials. Hence, considerable research attention has been directed towards developing strategies to control these factors in ZnO-based materials. For instance, methods such as high-temperature vacuum deposition methods, hydrothermal solution synthetic approach, as well as electrochemical deposition have been utilized to develop oriented ZnO nanorods, tubes, and porous membranes, respectively [38,39,40,41,42].

In recent times, nanostructured materials based on zinc oxide have garnered significant interest among biomaterial scientists and surgeons due to their distinct attributes, which confer upon them novel biological functionalities. Due to their nanoscale zinc oxide structure and the variety in their nanotechnology domains these composites have wide applications. Specifically in the recent bioenvironmental discoveries, a scientific team designed a Cs_2_O-Bi_2_O_3_-ZnO nanostructure that act as efficient sunlight-driven photocatalysts [43]. Furthermore, CuO-ZnO nanocomposites with varying Cu concentrations were synthesized by Zin Toe et al., using g the hydrothermal technique and cetyltrimethylammonium methacrylate (CTAM) as a surfactant. These nanocomposites were able to degrade organic pollutants via photocatalysis too [44]. In the latest bibliography, ZnO-based nanocomposites have various beneficial antibacterial properties against bacterial strains, such as *E. coli* [45], *S. enterica*, *S. paratyphi*, *S. aureus*, *K. pneumoniae* [46], *Bacillus subtilis* [47] and fungal strains, such as *A. flavus*, *A. nidulans*, *T. harzianum*, and *R. stolonifer* [48]. Notably, these structures can be very efficient not only in the diagnosis of tumors, but also in the treatment of them, since they are very effective as drug delivery systems [49]. Moreover, numerous studies conducted recently have suggested that ZnO nanoparticles may be useful in the treatment of inflammatory illnesses [50] and as theranostic materials, like in the study of Wu et al., where they created a nanomaterial by linking ZnO quantum dots to polyethylene glycol-based nanogel chains and growing metallic Au [51]. Since the biomedical applications of these nanocomposites are very extended this section will be described in depth below. Finally, the usage of nanomaterials in skin care products, including sunscreens, shampoos, creams, eye creams, and color cosmetics, has gained increased interested during the latest decade [49,52] and it is known that recently, nano-ZnO is extensively utilized in clinical studies and has various applications.

Notably, the remarkable electronic attributes exhibited by nano-ZnO structures render them appropriate for biosensor development. Moreover, their exceptional photoluminescent features, including adjustable emission wavelength, coupled with enhanced aqueous robustness and quantum yield, position ZnO-based quantum dots as promising bio-probes for cell and tissue imaging applications. Additionally, the exceptional catalytic and antimicrobial properties of ZnO nanostructures, combined with their biocompatibility, hold promise for diverse applications such as tissue regeneration, bacterial resistance, and wound dressing. For instance, the release of Zn^2+^ ions from nano-ZnO structures promotes bone formation in vitro [53], while simultaneously facilitates keratinocyte migration towards wound sites, thereby aiding in the healing process [54]. Moreover, the generation of reactive oxygen species (ROS) and Zn^2+^ ion release from zinc oxide nanoparticles can cause detrimental damage to bacterial cell membranes [55]. Furthermore, owing to its pH-responsive nature, ZnO can serve as a drug carrier in pH-responsive systems. Nonetheless, there exists controversy regarding the potential toxicity of nano-ZnO structures towards healthy tissue [56,57,58].

As a consequence of escalating environmental degradation, an aging populace, and the overuse of antibiotics, the incidence of ailments like osteoporotic nonunion, diabetes mellitus and diverse skin lesions has surged, alongside the emergence of antibiotic-resistant bacteria. This surge has spurred a significant requirement for biomaterials boasting novel and enhanced capabilities. Among these, nanostructured ZnO-based materials stand out as particularly promising for advancing the diagnosis and treatment of these ailments.

This study aims to comprehensively review up-to-date strides in the utilization of ZnO-based nanomaterials within the realm of biomedicine, with a specific focus on their applications in biosensing, tissue regeneration, bioimaging, drug delivery, as well as vaccines and immunotherapy. Furthermore, it elucidates various synthetic methodologies currently employed for the production of distinct ZnO nanostructures, alongside a comparative analysis of modification strategies. Lastly, this article delves into the toxicity mechanisms associated with ZnO nanoparticles, shedding light on the less-explored domain of nano-ZnO particles’ neurotoxicity.

## 2. Methodology

This literature review was conducted through different official databases including PubMed, Google Scholar, Elsevier and ScienceDirect, to identify relevant publications according to the topic. Various keywords have been used into those engines, such as “ZnO nanoparticles”, “ZnO nanocomposites”, “functionalized ZnO nanoparticles”, “ZnO nanocomposites in biomedicine”, “ZnO and biomedicine”, “Functionalized ZnO in biomedicine”, “ZnO for biosensors, vaccines, drug delivery and tissue regeneration”, “ZnO synthesis”, “ZnO Mechanochemical synthesis”, “ZnO Laser ablation”, “ZnO green synthesis”, etc. Also, a combination of those keywords was applied. Both original articles and reviews were studied. No limitation regarding an article’s publication date was set, but the most recent articles were preferred.

## 3. Synthetic Approaches for ZnO Nanoparticles’ Fabrication

The effectiveness of ZnO nanoparticles is primarily determined by factors such as surface properties, size distribution, particle morphology, and reactivity of its composition. Therefore, it is crucial to have ZnO nanoparticles with well-regulated structures that are consistent in both size and morphology for diverse biomedical applications. Various techniques have emerged for the controlled fabrication of ZnO nanoparticles in recent years, encompassing mechanochemical, hydrothermal sol–gel, as well as emulsion/microemulsion precipitation approaches, among others. Following the synthesis, the characterization of ZnO nanoparticles established on their inherent attributes is of utmost importance, and this is accomplished through the utilization of various available characterization techniques [34].

### 3.1. Mechanochemical Synthetic Approach

The mechanochemical approach offers a straightforward technique for the large-scale fabrication of ZnO nanoparticles. Utilizing ball mills induces an enhanced-energy influence on the precursor at decreased temperatures, resulting in the generation of small and uniform ZnO nanoparticles. Prolonged grinding times, however, may elevate the impurity levels. Noteworthy advantages of this method include minimal production costs, the attainment of fine and consistent particle sizes, as well as the development of a crystalline structure. In a study carried out by Nguyen et al. [59], the mechanochemical method was employed, in order to prepare ZnO nanoparticles characterized by an average size of 40 nm. Zinc acetate dihydrate and sodium hydroxide were utilized as primary constituents, with cetyltrimethylammonium bromide (CTAB) serving as a protective agent, followed by centrifugation and drying at 400 °C. The optimal milling time of 60 min resulted in ZnO nanoparticles possessing the smallest average size. The ensuing reactions are detailed below (Equations (1) and (2)):

(1) $$\mathrm{Zn}{\left({\mathrm{CH}}_{3}\mathrm{COO}\right)}_{2}+\mathrm{NaOH}\to 2{\mathrm{CH}}_{3}\mathrm{COONa}+\mathrm{Zn}{\left(\mathrm{OH}\right)}_{2}$$

(2) $$\mathrm{Zn}{\left(\mathrm{OH}\right)}_{2}\to \mathrm{ZnO}+H_{2}O$$

In a separate investigation, Aghababazadeh et al. [60] and Moballegh et al. [61] explored the impact of the temperature employed during heating on the size of the particles through crushing zinc chloride and sodium carbonate. More specifically, Aghababazadeh et al. achieved the synthesis of ZnO nanoparticles possessing particle sizes of ≈51 nm and specific surface area equal to 23 m^2^/g at 400 °C. Likewise, Moballegh et al. observed an increase in the particle size of ZnO from 27 nm to 56 nm upon increasing the heating temperature from 300 °C to 450 °C. Although this approach is facile, it is limited to large-scale synthesis, as it demands high-power consumption during the milling process. Mechanochemical synthetic approach includes laser ablation, as well as high-energy ball milling methods [35,62,63,64].

#### 3.1.1. Laser Ablation Approach

The laser ablation technique is a method that removes metallic ions from metal surfaces through the use of a laser beam, as well as a small quantity of liquids, such as CH_3_OH, CH_3_CH_2_OH, and distilled water, while the metal surface is immersed into the selected liquid. The approach is characterized by its ease and eco-friendliness, rendering it an effective and conveniently executable approach [65]. Nevertheless, there is a need to comprehensively understand and address the pyrolysis by-products that arise, especially when organic substances are present [66]. Noteworthy studies by Al-Dahash et al. [67] demonstrated the fabrication of spherical ZnO nanoparticles using laser ablation in sodium hydroxide aqueous solution ranging from 80.8 to 102.5 nm, approximately. Similarly, Farahani et al. [68] utilized laser ablation using a Zn target in a solution of CH_3_OH and distilled H_2_O to obtain ZnO nanoparticles (1–30 nm) characterized by almost spherical morphology. Additionally, Mintcheva et al. [65] reported the production of ZnO nanoparticles (40–100 nm) presenting a rod-like morphology with diameter equal to 30 nm.

Additionally, Thareja and Shukla [69] presented the fabrication of a colloidal suspension containing ZnO nanoparticles through pulsed laser ablation of a Zn metal target at ambient temperature in various liquid environments (water, isopropanol and acetone), albeit with a modest yield. When water and isopropanol were used as solvents, the result was spherical nanoparticles ranging in size from 14 nm to 20 nm. In the case of acetone, two distinct particle types were observed (a) spherical nanoparticles possessing size of ≈100 nm and (b) platelet-like structures measuring 1 μm in diameter and 40 nm in width.

Furthermore, Camarda et al. [70] synthesized crystalline ZnO nanoparticles utilizing ultra-short femtosecond pulsed laser ablation of a Zn plate in distilled water, characterized by an average size of approximately 30 nm.

Also, in a study by Gavrilenko et al. [71], ZnO nanoparticles were produced by utilizing a nanosecond pulsed laser to ablate a Zn target in both water and air environments, subsequently comparing their characteristics. The results revealed that the nanoparticles generated in the air exhibited a spherical shape, whereas those synthesized in water were larger and possessed a rod-like structure. Both samples exhibited the hexagonal wurtzite ZnO phase. However, due to interaction with nitrogen found in the surrounding atmosphere, the nanoparticles produced in the air contained a fraction of monoclinic zinc hydroxynitrate as well. A comparison between the ZnO nanoparticles produced in water and air indicated that the latter exhibited superior dispersibility in water.

#### 3.1.2. High-Energy Ball Milling Approach

The high-energy ball milling approach, conducted in an elevated shaker mill, constitutes a procedure for the fabrication of fine metal nanoparticles [72]. Its primary advantage lies in its capacity to produce large quantities of material simultaneously. However, drawbacks entail potential contamination deriving from milling balls and/or the environment, leading to inconsistently shaped nanoparticles [73].

ZnO nanoparticles were synthesized by Prommalikit et al. [74] through high-energy ball milling utilizing commercially available ZnO powder with an average particle size of 0.8 µm, resulting in particles with size ranging from 200 to 400 nm. Similarly, Mohammadi et al. [75] employed high-energy ball milling which led to the development of rod-shaped ZnO nanoparticles ranging from 20 nm to 90 nm.

### 3.2. Hydro-/Solvothermal Synthetic Approach

The hydrothermal synthesis of ZnO nanoparticles eliminates the need for natural solvents or additional treatments such as crushing and calcination, rendering the as-mentioned synthetic approach straightforward and environmentally friendly. This process takes place in an PTFE (polytetrafluoroethylene)-lined, Teflon-lined, or PPL (polyparaphenol)-lined autoclave. In this approach, a substrate mixture is continuously heated to a temperature ranging from 100 to 400 °C under high pressure (10–80 MPa) and subsequently remains undisturbed for a duration ranging from hours to days. At elevated temperatures and pressures, materials that are insoluble or resistant to dissolution can be effectively dissolved [76]. Various solvents, including water or organic solvents like ethanol or polyols, can be employed in these reactions, referred to as hydrothermal or solvothermal approach, respectively [77,78]. Hydrothermal methods not only ensure products’ enhanced purity and crystallinity but also provide regulation over the final nanostructure’s size, shape, as well as crystal phase depending on the composition mixture, processing temperature, and pressure [79,80,81], with minimal pollution because of the closed system environment [35,78]. However, drawbacks include the requirement for an expensive autoclave, limitations for studies due to the closed nature of the reactor, and potential safety hazards associated with the autoclave procedure [63,82,83].

Zhao et al. [84] employed a facile hydrothermal method to produce wire-like ZnO nanoparticles. In this approach, zinc acetate dihydrate, sodium dodecyl sulfate, PEG400, and ethanol served as precursors and were autoclaved for 16 h at 160 °C, followed by gradual cooling at ambient temperature. The resulting material was washed with ethanol to yield nanowires with a diameter of 30–50 nm. Another synthesis method for ZnO nanosheets was developed by Zheng et al. [85], who utilized the solvothermal method. In this process, an aqueous solution of Zn(NO_3_)_2_.6H_2_O, urea, and l-asparagine was autoclaved for 12 h at 180 °C and then cooled to ambient temperature. The as-received white precipitate was washed with ethanol, dried at 60 °C, and subsequently calcined at 300 °C for 2 h to obtain ZnO nanosheets.

Moreover, Bharti and Bharati [86] employed a hydrothermal approach to synthesize ZnO nanoparticles, achieving a length scale within the range of 15.8–25 nm and diverse morphologies. In a separate study, Wirunmongkol et al. [87] utilized an autoclave for the synthesis of ZnO nanoparticles, with NaOH and Zn(NO_3_)_2_.6H_2_O serving as precursors. The resulting nanoparticles exhibited shapes resembling tiny prisms and flowers, with dimensions ranging from 30 nm to 80 nm in width and 0.1–0.5 μm in length, dependent on the materials used for their fabrication.

Furthermore, Motelica et al. in their study compared ZnO nanoparticles synthesized through solvolysis using various alcohols, demonstrating that primary alcohols from methanol to 1-hexanol successfully yield ZnO synthesis, while secondary and tertiary alcohols do not, highlighting solvent importance. The nanoparticle shape varies based on the alcohol used, impacting properties like photocatalytic and antimicrobial activities, with ZnO obtained in 1-butanol showing the highest photocatalytic efficiency against methylene blue (MB) dye, suggesting potential customization for medical applications [88]. Also, members of the same team in 2023 investigated the synthesis of ZnO nanoparticles via forced solvolysis of zinc acetate dihydrate in alcohols with differing -OH group quantities, assessing the impact of alcohol type (n-butanol, ethylene glycol, and glycerin) on nanoparticle characteristics. It was observed that n-butanol yields smaller polyhedral ZnO nanoparticles (<30 nm), ethylene glycol produces rounded nanoparticles averaging 44 nm, and glycerin results in polycrystalline particles of 120 nm after water refluxing. Additionally, the article evaluates the nanoparticles’ photocatalytic activity against a mixture of methyl orange (MO), methylene blue (MB), and rhodamine B (RhB), demonstrating efficient degradation (>99.99%) over multiple cycles and strong inhibition of planktonic growth against both Gram-negative and Gram-positive bacterial strains, suggesting their potential for water purification and antibacterial applications [89].

### 3.3. Sol–Gel Synthetic Approach

ZnO nanoparticles’ synthesis through the sol–gel approach involves four stages: (a) hydrolysis of the chemical precursor, (b) polymerization, (c) gel formation, and (d) solvent evaporation through heat treatment [90,91]. The s–ol-gel method enables the production of ZnO nanoparticles in a finely powdered structure with a regulated chemical composition [90]. However, this technique comes with inherent limitations such as shrinkage, susceptibility to breakage during drying, and challenges in controlling porosity [92]. Despite these drawbacks, the simplicity of the protocol and the rapid generation of crucial materials often make it a frequently discussed topic in relevant literature and remains one of the most regularly employed approaches [93].

Kumar et al. [94] synthesized Mn-doped ZnO nanoparticles utilizing the sol–gel approach through the addition of isopropyl alcohol to zinc acetate dihydrate, subsequent to the addition of manganese acetate and urea under constant stirring until the complete evaporation of the solvent, leading to the formation of a uniform gel. Then, the gel was subjected to calcination at 500 °C, while the obtained powder was further calcined at 600 °C for 2 h to receive crystalline Mn-ZnO nanoparticles.

Also, Kumar et al. [95] employed the sol–gel technique utilizing zinc acetate, sodium hydroxide, and polyethylene glycol. The as-mentioned mixture was heated to 80 °C and then cooled to ambient temperature until the formation of a thick gel. Subsequently, the pH value was adjusted using HNO_3_ under constant stirring until a dark brown precipitate was formed, which was then dried at 80 °C. The dried powder was subjected to calcination at 800 °C for 2 h, resulting in the production of ZnO nanoparticles.

Within the framework of another study [96], zinc acetate dihydrate and ethanol were employed as solvents for synthesizing rod-shaped ZnO nanoparticles with size ranging from 81.3 nm to 85 nm, approximately.

### 3.4. Emulsion/Microemulsion Precipitation Synthetic Approach

Among various chemical methods, the microemulsion technique stands out as it is possible to effectively regulate the size and morphology of the produced nanoparticles. Microemulsions are created by water and oils, which are immiscible liquids and a surfactant, to stabilize them. The metal precursor is inside the water droplets, that are dispersed into the oil. Because the growth reaction happens only inside the droplets, the size is controlled and the nanoparticles do not agglomerate [97,98]. The advantages of this method encompass its simplicity, thermodynamic stability, and minimal accumulation. However, microemulsion techniques come with various drawbacks, such as the sensitivity of microemulsion stability to temperature and pH variations, along with the continual requirement for highly concentrated surfactants and/or cosurfactants that might cause irritation [63].

Ali et al. prepared vanadium-doped ZnO nanoparticles using the microemulsion precipitation process [99]. Microemulsions were formulated by combining equal moles of zinc acetate and vanadium chloride (VCl_2_) and then adding the CTAB (C_19_H_42_BrN) as a stabilizing agent. With temperatures ranging from 50 to 60 °C, the pH was raised to around 10, by adding ammonia. Then, the precipitation of a white product was achieved. To neutralize and eliminate organic impurities, the precipitates were washed multiple times using ethanol. After drying, the product was transferred to a 300 °C oven for 5 h, for calcination. XRD and SEM analyses confirmed small nanoparticles at 12 nm, with a crystalline wurtzite structure.

In their study, Emsaki et al. [100] employed the microemulsion technique to synthesize ZnO-ZnWO_4_ nanoparticles and tested them as photocatalysts in organic dye removal. The precursors, zinc (II) nitrate and sodium tungstate dehydrate, were dispersed in water. The organic phase was cyclohexane and two surfactants were used (polyoxyethylene lauryl ether and 1-butanol). Two microemulsions were created, one containing zinc nitrate (A) and the other containing NH_4_OH (B). The reaction was initiated by combining microemulsion (B) with microemulsion (A) and precipitating the product. The obtained precipitates underwent washing with an ethanol solution and then went through first a drying process and then calcination at 500 °C. The product was hexagonal-shaped ZnO-ZnWO_4_ nanoparticles, with a size range of 40–70 nm, according to the SEM results.

Furthermore, Pineda-Reyes et al. [98] conducted the synthesis of ZnO nanoparticles using the water-in-oil (*w*/*o*) microemulsion method. The organic phase was a combination of emu oil and surfactant (in a 1:1 ratio of Span 80:Tween 80) in various proportions. Subsequently, zinc acetate dispersed in water was added slowly under stirring. After a day of continuous stirring, NaOH 1 M solution, was added dropwise, to cause precipitation. The temperature was kept at 60 °C. After a few minutes under stirring at 1200 rpm, the precipitate was collected and washed with polar (water, acetone) and non-polar (hexane) solvents. After drying, the product was transferred for calcination at 800 °C.

Additionally, Wang et al. [101] produced 16 nm ZnO nanoparticles in microchannel reactor systems. Subsequently, the ZnO nanoparticles underwent a 2 h drying period at 130 °C and then calcination at 550 °C for 3 h, while Li et al. [102] similarly synthesized ZnO nanoparticles through a straightforward microemulsion procedure, resulting in varied morphologies such as columnar and spherical shapes.

### 3.5. Controlled Precipitation Synthetic Approach

The controlled precipitation method is widely employed for the production of ZnO nanoparticles, due to its ability to yield material with consistent properties, cost-effectiveness, and suitability for large-scale production without the need for sophisticated equipment. In this approach, the rapid and spontaneous reduction of a zinc salt solution occurs through a reducing agent, allowing for controlled particle growth with specified dimensions. The ZnO precursor is then precipitated in the solution, constituting a bottom-up method. Subsequent thermal treatment is applied to the precursor and then griding to produce fine powder. Key parameters in this synthesis encompass reagent concentrations, the ratio of substrate addition, and reaction temperature and pH [35,80].

Jeyasingh et al. [103] employed the controlled precipitation method to synthesize ZnO nanoparticles. The researchers utilized zinc acetate dihydrate, sodium hydroxide, and sodium chloride as precursors. The precipitation starts when the sodium hydroxide solution is added dropwise into the precursor solution. The white product is then collected and washed using a centrifuge. After drying at 90 °C for 5 h the product can be analyzed. The XRD analysis showed that the nanoparticles had a hexagonal wurtzite structure and the particle size was determined to be 41.6 nm.

Similarly, Jeyachitra et al. [104] proposed a synthesis method for ZnO nanoparticles with a different zinc precursor. Zinc sulfate heptahydrate (ZnSO_4_·7H_2_O) was chosen and sodium bicarbonate was used as a reducing agent. The methodology is the same. A solution of the reducing agent (0.1 M) is added dropwise into precursor’s suspension (0.1 M), which causes precipitation. The mixture is constantly under stirring. After removing impurities by washing with water, the precipitate is transferred to an oven for drying. The researchers used higher temperature for drying, than others (150 °C) and noted that during this stage, Zn(OH)_2_ is converted to zinc oxide nanoparticles. An average size of 30 nm and the expected hexagonal wurtzite structure were confirmed with XRD analysis.

### 3.6. Green Synthesis/Biosynthesis Approach

Green synthesis of nanoparticles involves the utilization of plant-based materials for biomedical applications, offering an environmentally friendly, cost-effective, biocompatible, and safe approach that enables the production of ZnO nanoparticles with minimal impurities. Nanoparticle synthesis through green methods demonstrates enhanced reactivity and efficiency compared to the use of expensive and hazardous chemicals. This approach encompasses the utilization of various plant components such as leaves, flowers, roots, seeds, and stems [105,106]. Extracts from these plant parts contain phytochemicals that serve as reducing agents and agents for stopping excessive growth or stabilizing nanoparticles at the same time [34,107]. Many plants are rich sources of phytochemicals, including phenolic compounds, nitrogen compounds, polysaccharides, vitamins, reducing sugars, terpenoids, alkaloids, and other antioxidant-rich metabolites [108,109,110,111]. Functional groups, such as hydroxyl, carboxylic, phenolic, and alkaloids, present in these compounds are responsible for their useful chemical properties, facilitating the reaction with metal salts resulting in zinc oxide nanoparticles with zero valence [112,113,114,115].

Typically, ZnO nanoparticles are synthesized through green methods utilizing extracts from plant materials. Umamaheswari et al. [116] introduced a variation to this conventional green synthesis approach by utilizing leaves from a Chinese radish plant. Ethanol was used to extract the compounds from the leaves. Subsequently, a mixture of 0.1 M zinc acetate (80 mL) and ethanol leaf extract (20 mL) was continuously agitated. Dropwise addition of 2 M NaOH with gentle stirring ensued to achieve an alkaline pH. Then, the temperature was raised to 70 °C for an hour followed by continued stirring using a magnetic stirrer for an additional 2 h. Through this process, white crystalline precipitate was formed, which was then washed with distilled H_2_O to neutralize the alkaline pH. XRD analysis confirmed the hexagonal wurtzite crystals forming ZnO nanoparticles, around 66 nm.

In another investigation by Bharath et al. [23], zinc oxide nanoparticles were synthesized using waste from palm trees. The phytochemicals were extracted with water after pulverizing the palm tree waste products. They used zinc nitrate hexahydrate as a precursor and ultra-sonification, followed by calcination at 400 °C for 1 h, resulting in light yellow nanoparticles. Analysis revealed the spherical shape of the ZnO nanoparticles with nanoscale dimensions of 30 nm.

Likewise, Alrajhi et al. [117] undertook green methods for the synthesis of ZnO nanomaterials utilizing aqueous extracts from *Salvia officinalis* leaves. The process involved blending the extract with a zinc precursor (zinc nitrate), hexamethylenetetramine and the hydrophilic linear polymer (PVA), followed by agitation for 60 min at 25 °C. The reaction was left at room temperature overnight and then the solids were collected with filtration and dried. The sizes and shapes of the nanostructures, produced with this method, were not uniform. Selim et al. [118] produced ZnO nanoparticles with extract from flowers of *Deverra tortuosa*. The cold maceration process was employed to obtain the phytochemicals from the flowers. Then, the zinc precursor was added upon moderate heating. Precipitation occurred after a day. Subsequently, the precipitate was washed with ethanol and water and dried. Lastly, the product was calcinated at 400 °C for 2 h. X-ray diffraction confirmed the usual hexagonal structure of the zinc oxide nanoparticles. The size of the nanoparticles was estimated at a range of 9–30 nm with HRTEM.

Additionally, numerous scientists worldwide have adopted similar or modified versions of this green strategy for ZnO nanoparticles’ synthesis. This eco-friendly, readily available technique holds promise for large-scale production of ZnO nanoparticles, with extensive avenues for future exploration. Similarly, many microorganisms serve as bio-reducing agents for ZnO nanoparticle preparation, a method known as microbial synthesis.

The microbial synthesis method shows promise for ZnO nanoparticles’ production due to its efficiency, environmentally friendly nature, cost-effectiveness, simplicity, and potential for large-scale manufacturing, enabling the production of commercially viable, biocompatible nano-ZnO particles. Unlike plants, microbes offer advantages in terms of availability and reproducibility [34,119]. A variety of biological organisms, such as bacteria, yeast, fungi, viruses, and algae, participate in this process, leveraging various biomolecules including enzymes, proteins, amino acids, polysaccharides, lipids, and vitamins. These biomolecules serve as bio-reducing agents, capping agents, and facilitators of nanostructure growth [34,120]. The complex biological mechanisms that result in nanoparticle production are still not well understood, prompting scientists to focus on elucidating the biological processes involved. Some studies suggest that NADH (Nicotinamide Adenine Dinucleotide) and NADPH (Nicotinamide Adenine Dinucleotide Phosphate)-dependent nitrate reductase enzymes play crucial roles in metal nanoparticle creation [121,122].

**Figure 1:**
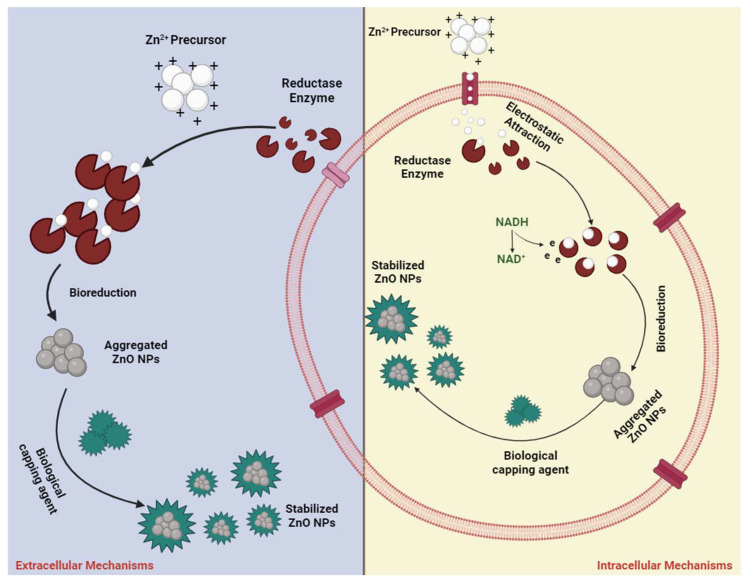
Schematic illustration of microbe-mediated intracellular, as well as extracellular synthetic mechanisms of ZnO nanoparticles (ZnO NPs) [127].

The microbial production of zinc oxide nanoparticles necessitates the presence of Zn precursors, typically provided as soluble salts. Upon introducing these precursors to the cells, ZnO nanoparticles precipitate. The required time can range from a few minutes to a few hours, depending on the conditions. The precursor ions are firstly transferred to the cells by electrostatic forces and enter the cell (Figure 1). Within the cell wall, initiation of nuclei formation takes place by enzymes, as well as nanoparticles’ development within the cytoplasm. At the same time, enzymes that are excreted by the cells, function as reducing agents, thereby enabling nanoparticles’ formation and proliferation. Microbes also excrete proteins that control the nucleation, limiting the nanoparticles’ size. The visual confirmation of ZnO nanoparticles’ production often involves white deposition in the culture container or a cloudy suspension [123,124,125,126].

The Gram-negative cyanobacterium *Arthrospira platensis*, has been used for ZnO nanoparticle synthesis [128]. The strain was incubated using Zarrouk’s media at a temperature of 30 °C, with ZnO nanoparticles being produced during the logarithmic phase of microbial growth. The culture underwent centrifugation to separate the biomass, which was then washed distilled water was added. This mixture was allowed to stand for 48 h before undergoing another round of centrifugation to collect the supernatant. Subsequently, zinc acetate dihydrate was added to the supernatant. After 24 h at a shaking incubator, with the temperature set to 30 °C, the precipitate was collected and dried. The ZnO nanoparticles were observed with TEM with sizes ranging from 30–55 nm.

An aqueous extract from the microalgae *Chlorella* has been used by Khalafi et al. to synthesize nanoparticles [129]. The precursor (zinc acetate) was mixed with the extract and heated at 60 °C. The experiment resulted in the formation of ZnO nanoparticles of 22 nm in size, confirmed with TEM and XRD.

Sanaeimehr et al. used *Sargassum muticum* algae extract in the biosynthesis of ZnO nanoparticles. The extract is used with a zinc precursor and heated at 70 °C for 4 h. After collecting the product, it is dried and then put at 450 °C for 4 h [130]. Additionally, another study focused on the preparation of ZnO nanoparticles using a modified version of the co-precipitation method with bacterial extract [131]. A modified method of biological extraction was used to create the extract. The extract was used in the form of a powder which was added into the mixture of the precursor and sodium hydroxide, causing precipitation. The product was then collected, washed and dried. The nanoparticles possessed a wurtzite crystalline phase and a size around 35 nm.

The tolerance of these microorganisms to the presence of heavy metals, is a crucial factor for their potential in nanomaterials’ biosynthesis. Many different microorganisms, particularly bacteria, have been utilized for synthesizing ZnO nanoparticles, with bacteria being favored over other eukaryotic microorganisms, due to their easily manipulated genome. The bioproduction of ZnO nanoparticles has been researched using bacteria including *Lactobacillus sporogen*, *Streptomyces* sp., *Lactobacillus paracasei*, and *Bacillus megaterium* [34,120].

ZnO nanoparticles synthesized with many different methods are being investigated for a range of applications. For instance, researchers have examined the effects of *Bacillus megaterium*-mediated ZnO nanoparticles on metabolic activity, membrane integrity, intracellular ROS generation, and apoptosis induction. The toxicity of these ZnO nanoparticles was evaluated at doses ranging from 3.12 to 100 mg/mL in human mesenchymal stem cells over a 24 h period. Metabolic activity of treated cells was assessed using Alamar blue testing, while FDA (Fluorescein Diacetate) staining was employed to qualitatively measure cellular effects and morphological changes induced by the ZnO nanoparticles [132]. Similarly, another study investigated the bacteriostatic effect of these nanoparticles on drug-resistant microbes, such as methicillin-sensitive *Staphylococcus aureus* and *Acetinobacter baumannii*, identifying carboxyl and amid groups as key participants in the biosynthesis process of zinc oxide nanostructures [133].

Additionally, Singh et al. reported on the antioxidant properties of ZnO nanoparticles and *Pseudomonas aeruginosa* rhamnolipid-stabilized nanoparticles. Their findings suggested that ZnO nanoparticles stabilized by rhamnolipid exhibited spherical nanoparticle formation, with sizes ranging from 27 to 81 nm, attributed to the long carbon chains of rhamnolipids serving as effective capping agents and hindering micelle aggregation on carboxymethyl cellulose surfaces [134].

Furthermore, the extracellular synthesis of nanoparticles from fungi offers significant advantages in terms of scalability, downstream processing, and cost-effectiveness. Fungal strains are preferred over bacteria, due to their greater resilience and ability to accumulate metals. A study focusing on the anticancer potential of *Xylaria acuta*-mediated zinc oxide nanoparticles revealed their potent antiproliferative effects against human MDA-MB 134 mammary gland carcinoma cells [135].

Similarly, another investigation explored the use of nano-ZnO particles to target bacterial biofilms, demonstrating their ability to inhibit biofilm formation by various microbes, including *Staphylococcus aureus* and *Pseudomonas aeruginosa* [136]. Different fungal strains exhibit variations in structure, shape and size, resulting in different functionality, and by extension different potential applications.

An experiment evaluating the effect of changing fungi on nano-ZnO particles’ synthesis revealed distinct crystalline structures and differing cytotoxic and antibacterial activities for each nanoparticle. The study concluded that nanoparticle shape and size are crucial factors for biomedical applications.

Comparably, micro and macroalgae show promise in synthesizing metal nanoparticles [137]. Both unicellular and multicellular photosynthetic algae have been tested for metal nanoparticle synthesis. Previous studies have focused on gold and silver, but ZnO nanoparticles have started to be synthesized by researchers, with the added benefit of their ability to degrade toxic chemicals [138], making them particularly noteworthy. Various algae species, including *Chlorella* [139], *Sargassum muticum* [140], *Cladophora glomerata* [134], and *Padina gymnospora* [141], have been utilized by researchers for ZnO nanoparticles’ fabrication.

### 3.7. Other Synthetic Approaches

Several chemical methods, like chemical vapor deposition, spray pyrolysis and microwave-assisted synthesis, can be used to synthesize ZnO nanoparticles. ZnO nanoparticles with a carbon coating, were produced by researchers using chemical vapor deposition technique [142]. Camphor was used as a carbon precursor and zinc acetate as the zinc precursor. After 30 min at 450 °C in a muffle furnace, the reactants were grinded. Then, the reactants were put in different containers and transferred to a furnace with N_2_ flow, at 750 °C. After 3 h, the nanoparticles have been formed. A similar technique was utilized by Choi et al. [143] to synthesize two-dimensional nanomaterials. The furnace was set between 700 °C and 900 °C and Ar gas provided flow. Graphite and ZnO powder were the precursors of the as-produced nanomaterial.

Another technique that has been used to synthesize nanoparticles is spray pyrolysis. The process involves spraying a solution to a heated surface thus creating a thin film. Antibacterial thin films of Al-doped ZnO were created using this method by Manoharan et al. [144]. Glass substrates were used and the temperatures used reached 400 °C. The spray head continuously scanned the surface at a steady speed, achieving uniform film growth. Similarly, Srinivasulu et al. employed this approach to coat corning glass substrates with zinc oxide films, doped with iron. This investigation demonstrated that the magnetic and physical characteristics of the product could be controlled by changing the concentration of the ferric chloride in the mixture [145].

Microwaves have also been used often in the synthesis of nanomaterials. The reactants are heated rapidly and uniformly with this method, resulting in nanoparticle fabrication. Common precursors like zinc nitrate dehydrate, have been tested with this method. After ten minutes in a microwave at 750 W, single crystalline ZnO nanostructures were formed, as reported by Salah et al. [146]. Other structures like nanorods have been reported using this method and zinc nitrate as a precursor by Sadhukhan et al. The products have a potential for biomedical applications, such as antibacterial, anticancer and bioimaging [147]. These nanorods can be conjugated with pharmaceutical substances to enhance their properties. In the thorough literature search of Wojnarowicz et al., a discussion about the preparation of ZnO by the microwave method is conducted. Advances in the microwave solvothermal synthesis have made it possible to obtain ZnO with an adjustable particle size in the range of approximately 20 nm to 120 nm [148].

Nevertheless, a significant drawback of numerous existing methods lies in their high cost, attributed to expensive chemicals, instruments, and substantial energy requirements. Moreover, the utilization of toxic compounds is dangerous for the environment.

Several studies affirm that the chemical synthesis of nanoparticles is not only toxic but also lacks biocompatibility, limiting its applicability in biomedical and agricultural contexts [149]. Consequently, alternative approaches, such as green synthesis and biological synthesis have been developed to mitigate the negative effects of the chemical synthetic methodologies.

## 4. Modification Approaches of ZnO Nanoparticles

The exploration of potential applications for ZnO nanoparticles includes efforts to improve the characteristics of the nanoparticles by reducing agglomeration, enhance targeted delivery, and provide synergistic effects for biomedical applications. To achieve these goals, nano-ZnO particles are often conjugated with various materials such as polymers, medicines, zero dimensional nanomaterials like carbon quantum dots, and other metal nanoparticles. Conjugation methods involve surface modification of the nanoparticles or entrapping them within polymer nanostructures or nanoparticles, either during or after nanoparticle fabrication [35,150]. For instance, surface modification of ZnO nanoparticles using ethanethioamide (C_2_H_5_NS) has been shown to improve the particles’ antibacterial bioactivity, resulting in the formation of a floral-like structure when ethanethioamide is mixed with the metal salt at the first stage of the reaction [151]. Additionally, researchers employed vinyl triethoxysilane (VTES) to prepare surface-modified ZnO nanoparticles, leading to reduced particle aggregation and slightly larger crystalline structures compared to pure nanoparticles [152].

Others focused on modifying nano-ZnO particles with calcium, utilizing calcium nitrate to produce Ca-doped nanoparticles for use as nanosensors capable of detecting trace amounts of elements, including pharmaceutical drugs in urine. The doped particles exhibited similar crystalline structures to unmodified ones, with calcium doping enhancing the electrochemical properties of the nanomaterial, facilitating the detection of trace elements.

Furthermore, researchers created ZnFe_2_O_4_ nanosheets that could be used to detect trimethylamine in gases [153,154]. The modification with FeSO_4_ was performed on pure ZnO nanoparticles to create ZnFe_2_O_4_ nanosheets with reduced aggregation and improved porosity, facilitating gas entrapment and detection [85].

Similarly, efforts to enhance the antibacterial properties of ZnO nanomaterials through various modifications have been reported. Researchers created nanocomposites by combining zinc oxide nanoparticles and curcumin particles ranging from nano to micro size. The goal was to create a synergy between the different particles and enhance the antibacterial properties. ZnO’s crystallinity was not affected, as was indicated through XRD analysis, while the antibacterial effect was improved [155].

Surface doping with manganese has also been tested and resulted in enhanced bactericidal properties. The one-pot co-precipitation method that was used resulted in similar crystalline structures as the pure nanoparticles [103]. Likewise, a thin polymer film was created by anchoring folic acid doped-ZnO nanoparticles into poly(vinyl alcohol). This nanocomposite exhibited hydrophilic properties, which could be adjusted by varying the doped nanoparticle presence on the thin film. The nanoparticles were uniformly distributed on the polymer film, leading to improved antibacterial effects compared simple nanoparticles that were not embedded on a matrix, as demonstrated through bactericidal assays [156].

Another useful nanocomposite can be produced through the incorporation of magnetic iron oxide (Fe_3_O_4_) nanoparticles alongside zinc oxide nanoparticles. Bisht et al. modified the Fe_3_O_4_ nanoparticles first by coating them with trisodium citrate. The resulting crystal structure closely resembled that of the unmodified ZnO nanoparticles. The properties of the composite were superior from the properties exhibited by the different parts when used in isolation, exhibiting superior magnetic properties and bactericidal properties [157]. Metal oxide nanoparticles possess significant electrocatalytic potential, large specific surface area, high surface reactivity, and conductivity, rendering them ideal for manufacturing electrochemical sensors for drug detection in bodily fluids [158].

Researchers used 1-butyl-3-methylimidazolium tetrafluoroborate (C_8_H_15_BF_4_N_2_) to modify zinc oxide nanoparticles produced via a green synthetic approach to create a cathode. The nanocomposite cathode exhibited enhanced sensing capabilities compared to conventional cathodes when used with the micro-auto lab system to detect two anticancer drugs in urine or blood [158].

Using different molecule, modifications can be made, thus, ZnO nanoparticles become more versatile for application across diverse fields, including that of biomedicine.

## 5. Biological Applications of Nanostructured ZnO

Zinc oxide nanocomposites have garnered significant attention in the realm of biological applications due to their unique properties and versatile characteristics. The inherent biocompatibility of ZnO makes it an attractive candidate for diverse biomedical purposes, such as drug delivery, imaging and biosensing [49,159,160]. The high surface area-to-volume ratio of ZnO nanocomposites allows efficient drug loading, enabling targeted and controlled release for therapeutic applications.

**Figure 2:**
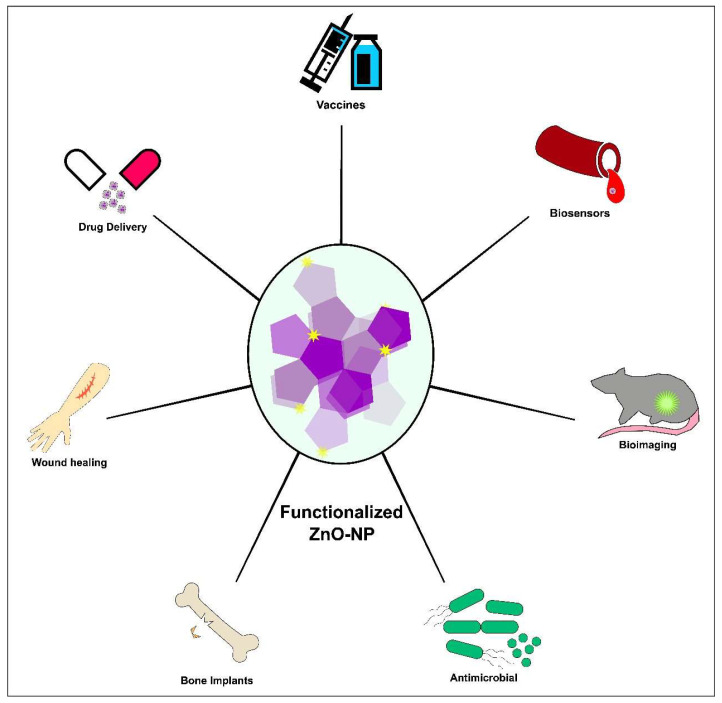
Schematic illustration of biological applications of functionalized ZnO nanoparticles (ZnO NPs).

Additionally, nano-ZnO particles exhibit inherent antimicrobial properties, making them effective for against a broad range of pathogens and enhancing their utility in antibacterial and antifungal applications [24]. The photocatalytic activity of ZnO contributes to its use in photodynamic therapy. Furthermore, the ability of ZnO nanocomposites to be functionalized with biomolecules and exhibit tunable surface properties enhances their compatibility with biological systems [160]. This collective set of attributes positions render ZnO nanocomposites promising materials for advancing biotechnological and biomedical applications, supported by a growing body of research and publications in the field (Figure 2).

### 5.1. ZnO-Based Biosensors

A biosensor system enables the quantitative measurement of specific parameters during complex biochemical reactions using a transducer linked to a biologically derived recognition entity [161]. The accurate and timely detection of diseases, their diagnosis and data assimilation play a crucial role in effective health monitoring.

Biological nanosensors can achieve this by monitoring levels of cholesterol, glucose, and urea in blood serum. Additionally, they can detect CO_2_ and ethanol vapors in in breath and identify pathogens such as bacteria and viruses in the air [162]. A biosensor has the capability to directly sense these targets and transduce the signal into an electrical or optical measurable form. In certain instances, biological components, such as enzymes, are integrated into a transducer, like a ZnO nanostructure, to generate an electronic/optical output signal [163].

#### 5.1.1. Non-Enzymatic Biosensors

While ZnO nanomaterials are highly desirable for immobilizing low isoelectric enzymes in the construction of enzymatic based biosensors, they are not considered suitable for non-enzymatic biosensor fabrication due to their limited catalytic activity through biomolecules. Consequently, there is a need to modify the surfaces of ZnO nanostructures by incorporating a nanostructured transition metal oxide catalyst such as TiO_2_, Fe_2_O_3_, CuO, etc., or to create a nanocomposite of ZnO and metal oxide to enhance the catalytic capability for the target analyte [164,165,166].

Wang et al. conducted a study where they utilized an electrochemical deposition method to synthesize ZnO nanorods (NRs) arrays on an indium tin oxide (ITO) substrate, aiming to create a highly sensitive sensor for detecting H_2_O_2_ levels. The research revealed the presence of an optimal sintering temperature within a specific range, crucial for enhancing the crystalline quality and carrier mobility of ZnO nanorods. The research results indicated that a sintering temperature of 400 °C was identified as the optimal condition. This discovery highlighted that subjecting the ZnO nanorod array-modified electrode to high-temperature sintering significantly enhanced both the intensity and stability of the photocurrent. As a result, the modified electrode demonstrated heightened sensitivity and exhibited a linear response when detecting H_2_O_2_ [167].

In their research, Zhou et al. employed the electrospinning technique to coat fluorine-doped tin oxide (FTO) with ZnO-CuO hierarchical nanocomposites. The FTO substrate had a porous and three-dimensional structure and this approach was applied for the construction of non-enzymatic biosensors. The ZnO-CuO nanocomposite/FTO exhibited enhanced non-enzymatic biosensing capabilities, with a notably improved sensitivity of 3066.4 μA mM^−1^cm^−2^ and a low detection limit of 0.21 μM. Despite a limited linear detection range, the electrospinning method employed for electrode fabrication ensured reproducibility and stability. The superior sensing performance was attributed to the three-dimensional porous morphology, providing a high surface area [168].

In 2016, Palasinamy et al. developed a hydrogen peroxide sensor using a graphene oxide (RGO)/ZnO composite, created through the simultaneous electrodeposition of ZnO and electrochemical reduction of graphene oxide (GO). The flower-like ZnO nanostructure microstructures, anchored near the RGO surface area, forming interconnected branch networks with the RGO sheets. This eco-friendly approach preserved the original morphology of nano-ZnO and the wrinkled structure of RGO even after 80 °C heat treatment, displaying excellent thermal stability. The composite demonstrated high electrocatalytic activity due to the large surface area, enhancing analyte diffusion efficiency. The RGO/ZnO composite film exhibited an extremely rapid response to H_2_O_2_, indicating a swift catalytic reduction process. The sensor showed high sensitivity (13.49 µA µM^−1^cm^−2^) with a broad linear detection range (1.0 to 22.48 µM) and a stable response to H_2_O_2_ [169].

#### 5.1.2. Glucose Biosensors

Glucose plays a vital role as a metabolite for living organisms, especially for individuals dealing with diabetes. Imbalances in glucose metabolism, often stemming from insulin deficiency and hyperglycemia lead to abnormal blood glucose levels [170]. Among various glucose monitoring methods, glucose biosensors offer significant advantages, including rapid response, high sensitivity, and stability. These attributes have sparked extensive and intriguing research into innovative detection strategies for glucose biosensors [171].

In the study by Wu and Yin, a highly sensitive glucose sensor was created using ZnO-CuO composite nanofibers fabricated through electrospinning and optimized thermal treatment. The CuO-ZnO modified Pt electrode, employed in amperometric glucose detection at low potentials, showed exceptional performance with a detection limit of 0.126 μM, a broad dynamic range (8.00 × 10^−7^ to 3.8 × 10^−3^ M), and remarkable sensitivity of 463.7 μA/(mM·cm^2^). The modified electrode also exhibited superior electrocatalytic activity, high sensitivity and rapid amperometric sensing for glucose oxidation, along with good selectivity, long term stability and excellent reproducibility. These findings offer a valuable foundation for advancing non-enzymatic glucose sensor development [172].

SoYoon et al. introduced a novel architecture composed of CuO nanoleaf and ZnO nanorods for a non-enzymatic glucose sensor. Amperometric response data demonstrated that the Cu/CuO/ZnO hybrid electrode exhibited a favorable response to glucose within a linear range of 0.1 mM to 1 mM, with a sensitivity of 408 μA mM^−1^ cm^2^ and a low detection limit of 18 μM. The CuO nanoleaf/ZnO nanorods structure displayed heightened electrocatalytic activity in glucose oxidation, attributed to the lower over-potential and larger electroactive surface area of the CuO nanoleaf structure and the one-dimensional ZnO nanorod structure [173].

Marie et al. in 2018, cultivated ZnO nanorods on FTO electrodes and incorporated Fe_2_O_3_ to enhance their performance as a non-enzymatic glucose sensor. The fabrication process involved a straightforward solution route for ZnO nanorods growth, followed by a surface dip-coating method to functionalize it with Fe_2_O_3_ [174].

In the study bvy Jung et al. a flexible field effect transistor glucose sensor utilizing nickel oxide quantum dots (NiO QDs) on zinc oxide nanorods was developed. The ZnO nanorods surfaces were enhanced with NiO QDs through radio frequency magnetron sputtering, improving electrocatalytic features and surface area. The non-enzymatic glucose sensor demonstrated excellent selectivity, stability, and repeatability in glucose detection. The sensor showed efficient glucose concentration measurement in human whole blood and serum samples, showcasing its potential for applications in clinical and non-clinical fields [175].

#### 5.1.3. Enzymatic Biosensors

Lately, for the identification of particular substances, ZnO nanomaterials modified with metal/metal oxide have been additionally customized with enzymes.

In the research of Lu et al., a simple and environmentally friendly hydrothermal method was employed to synthesize nanosized flower-like ZnO, offering a convenient and cost-effective process. The ZnO/chitosan composite matrix was formed by dispersing ZnO into a chitosan solution, combining the benefits of inorganic and organic polymer components. Biosensor’s fabrication and experimental conditions were optimized resulting in a H_2_O_2_ biosensor with a rapid response under 5 s. The sensor exhibited favorable reproducibility and stability, retaining approximately 78% of its original response [176].

Ahmad et al. developed a calcium sensor using a highly conductive seed layer. They established this layer on the substrate by sequentially depositing a ZnO seed layer, Ag nanowire, and another ZnO seed layer, forming a sandwich-like structure. Electrochemical characterizations were conducted to assess the role of each material, identifying the significant role of Ag nanowires in enhancing device’s sensitivity. This study introduces a valuable strategy for improving sensing performance offering insights applicable to the design of other biosensors with enhanced capabilities [177]. Table 1 summarizes the main information obtained from the aforementioned studies.

### 5.2. Bioimaging

ZnO quantum dots (QDs) produced through conventional methods exhibit several drawbacks, including a low quantum yield and a broad photoluminescence band. Additionally, ZnO QDs face instability in aqueous solutions due to water exchanging with the organic protective groups on their surface, leading to the destruction of luminescent centers and aggregation, consequently quenching their fluorescence. This challenge can be addressed through surface modification using specific ligands, such as polyethylene glycol methyl ether (PEGME), PEG(COOH)_2_, polyvinylpyrrolidone (PVP), and oleic acid (OA) in conjunction with diethanolamine (DEA) [160].

In 2023, Wanas et al. developed a graphene/folic acid-zinc oxide (GN/FA-ZnO) nanocomposite for dual mode emissions in cancer bioimaging applications. The nanocomposite exhibits low toxicity, high photostability, and dual-mode emissions that provide long luminescence lifetime and multicolor emissions visible to the naked eye. In vivo bioimaging experiments on mice with Ehrlich tumor implants demonstrate clear fluorescence, confirming the targeting behavior and selective bioimaging capability of the of the GN/FA-ZnO nanocomposite [178].

Hing et al. synthesized nanowires with green fluorescence, demonstrating their applicability for targeted imaging of cancer cells. Labeled with ^64^Cu for PET imaging in normal mice, the biodistribution revealed that non-targeted ZnO nanowires primarily accumulated in liver. The fluorescence of ZnO nanowires could be enhanced for cancer-targeted optical imaging through surface functionalization, improving water solubility, biocompatibility and reducing cellular toxicity. Additionally, red fluorescent nano-ZnO particles were developed by conjugating ^64^Cu and TRC105 to nano-ZnO particles, exhibiting tumor-targeted capabilities. PET imaging demonstrated high radioactivity accumulation in the liver, tumor and abdominal area, emphasizing effective tumor targeting and image contrast. The study concluded that TRC105 conjugation played a significant role in the increased uptake of nano-ZnO particles in the tumor, presenting promising applications in cancer imaging [26].

Moreover, Xiong et al. employed a two-step polymerization process to fabricate luminescent nano ZnO@poly(MAA-co-PEGMEMA) hybrids with tunable photoluminescence for cell imaging. Cytotoxicity tests revealed high cell viability, with over 90% survival when the concentration of ZnO QDs were below 0.2 mg mL^−1^. The ZnO QDs exhibited stable luminescence during cell culturing, while distinct emissions in the green and yellow ranges. This study highlights the potential of these stabilized ZnO QDs for bioimaging applications, offering controlled photoluminescence and minimal toxicity [179].

In research performed in 2013, nanoparticles were successfully synthesized using NaOH, KOH and LiOH as bases, resulting in varied colors as blue, green and cyan and orange. The research highlights LiOH as the most efficient base for controlling ZnO nanoparticle emission properties, with varying LiOH concentrations [180].

### 5.3. ZnO Nanostructures for Tissue Regeneration

#### 5.3.1. Antimicrobial Properties

ZnO nanoparticles showcase impressive potential as antimicrobial agents against a plethora of pathogens. Zinc oxide acts in a bactericidal [181], as well as fungicidal [182] manner, meaning that it not only pauses the proliferation of the pathogen, but also restricts their ability to reestablish active colonies. In the study by Cierech et al., the antifungal properties of zinc oxide nanoparticles against *Candida albicans* and their integration into polymethyl methacrylate (PMMA) resin material. Nanopowder with an average particle size of 30 nm was obtained, and ultrasound activation reduced the size of ZnO conglomerates in the acrylic resin solution by 11 times, indicating successful incorporation of ZnO NPs, necessitating further investigation to improve composite homogeneity and assess microbiological properties, strength, and biocompatibility for potential clinical use [183].

The exact mechanisms through which such effect is achieved remain yet to be determined. However, there are several studies in the past years that investigate such properties and mechanistic actions and so far, they have shown that ZnO antimicrobial properties act through (a) targeting the bacterial membrane causing disruptions and cell death [184], (b) targeted enhanced ROS production in bacterial cells through photocatalysis [185,186], or (c) Zn^2+^ release, that cause damage to important macromolecules (DNA, proteins) and mechanisms. These beneficial effects are highly dependent on the nanoparticles’ concentration as well as the size. Studies have proven that the efficacy of ZnO’s antimicrobial properties is directly correlated to the nanoparticles’ size, where the smaller the more effective [187].

#### 5.3.2. ZnO-Based Nanostructures for Wound Healing and Bone Implants

Electrical stimulation of cells has shown great potential in wound healing and tissue repair [188] through activation of different mechanisms and pathways, resulting in enhanced cell proliferation, migration, and differentiation. Attempts were made in order to take advantage of these effects in a targeted manner; hence the piezoelectric properties of ZnO-based nanomaterials have been employed, creating a self-activating source, inside the tissue, producing electric fields through non-invasive and selective means [189,190].

Radwan-Pragłowska et al., in their recent work from 2020, studied hybrid bilayer PLA/chitosan nanofibrous scaffolds doped with different nanomaterials, including ZnO, where they employed its piezoelectric properties for cell stimulation and application in burn wound healing [191].

Moreover, ZnO and biomacromolecules have been combined in various formulations to enhance wound healing. Blinov et al. developed gels by combining nano and micro ZnO with biomacromolecules, including maltodextrin, agar-agar, methylcellulose, amylopectin, and hydroxyethyl cellulose. Hydroxyethylcellulose was identified as the optimal choice for gel preparation and was applied in treating burns on mongrel white rats, either alone or in conjugation with ZnO. The study revealed that the addition of micro-ZnO to a hydroxyethylcellulose gel increased the burn healing rate in rats by 16.23% compared to the hydroxyethylcellulose gel alone [192].

Raguvaran et al. showcased that incorporating nanostructured zinc oxide into hydrogels made of sodium alginate and gum acacia significantly reduced cytotoxicity compared to the standalone zinc oxide, all while maintaining effective wound-healing characteristics. The hydrogel matrix played a crucial role in minimizing direct contact between nano-ZnO’s cells, thereby controlling the release of Zn^2+^ and thus, addressing concerns regarding the potential toxicity associated with nanostructured zinc oxide [193].

In the study of Balaura et al. it was introduced a novel wound dressing made of collagen and essential oil-functionalized ZnO nanoparticles, aiming to enhance burn treatment and prevent wound sepsis in burn or chronic wound patients. Physico-chemical characterization involved Fourier-transform infrared spectroscopy (FTIR) and scanning electron microscopy (SEM), while in vitro biocompatibility, cytotoxicity, and antimicrobial potential were assessed on human fibroblast cells and bacterial models (*S. aureus* and *E. coli*), and in vivo studies were conducted on a rat burn wound model. The functionalized ZnO nanoparticles, sized 15–20 nm with approximately 1% orange essential oil, demonstrated grain-like shapes and minimal aggregation, showing no toxicity in vitro, and the wound dressings displayed promising bioresorbable scaffold properties with excellent biocompatibility and antibacterial activity, making them potentially valuable for burn patient wound care [194].

Rakhshaei and Namazi developed a nanocomposite hydrogel using carboxymethyl cellulose, incorporating zinc oxide loaded MCM-41 mesoporous silica and tetracycline, a broad-spectrum antibiotic. The inclusion of nano-ZnO not only enhanced antimicrobial properties against *E. coli* but also underwent a cytotoxicity assessment with adipose tissue-derived stem cells revealed 63% cell viability after 24 h for the nano-ZnO containing hydrogels contrast to 81% in hydrogels without ZnO. Nevertheless, cell viability showed a continuous increase in the following days in both cases [195].

ZnO has also seen use in bone healing/bone implant fields since it is a fundamental element for bone formation and preservation. The use of zinc in bone-specific application can stimulate bone-related growth factors that initiate and assist in bone formation. In 2023, Nekounam et al. integrated various concentrations of nano-ZnO particles into cellulose nanofiber matrices through electrospinning. Analyses including XRD, FTIR and Raman spectroscopy indicated improved structure formation with lower defect density in the presence of nano-ZnO particles. Cellular cytotoxicity assays demonstrated the excellent biocompatibility of nano-ZnO-CNF composites and MG-63 cells exhibited robust attachment and spreading on the surface of all nanocomposites. This study provides valuable insights into nano-ZnO particles-incorporated CNF, suggesting their suitability for applications in bone tissue engineering [196].

The study by Amna et al. employs electrospinning to produce industrial scalable one-dimensional ZnO-doped TiO_2_ nanofibers. The nanocomposite, prepared through colloidal gel synthesis exhibits high purity and crystalline nature. Cell proliferation assay and microscopy observations demonstrate enhanced adhesion, proliferation and spreading of C2C12 cells on ZnO/TiO_2_ nanofibers in tissue engineering, benefitting from the technology’s scalability, cost-effectiveness, reproducibility and high-throughput capabilities for optimizing cell-nanofiber systems [197].

In a study of a scientific team in 2019, the aim was to improve composite resins using Ag-doped ZnO nanoparticles and evaluate their antibacterial and mechanical properties against 7-day *Streptococcus mutans* biofilm. Synthesized via polymeric precursor and co-precipitation methods, the ZnO/Ag NPs showed better biofilm inhibition with nanospheres than nanoplates, suggesting the potential of this material as a new restorative option as long as for dental implant [198].

Of great significance in tissue engineering and regeneration is the formation of new blood vessels. Studies have shown that reactive oxygen species production induces the expression of specific molecules that, in turn, promote angiogenesis.

**Figure 3:**
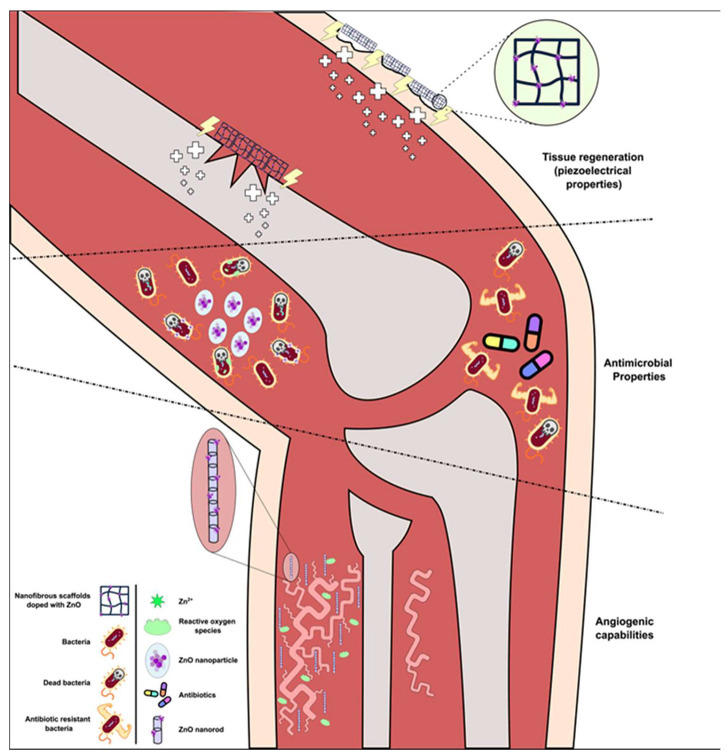
ZnO functionalized nanocomposites for application in tissue generation and healing.

Hassan et al. investigated, in their study during 2021 [199], the effect of ZnO nanorods (NRs) in wound healing acceleration and angiogenesis promotion through ROS production. However, they discovered that high concentrations of ZnO nanorods resulted in decreased angiogenic effect due to the elevated concentration of ROS, which resulted in cell damage rather. Hence, there is a point of equilibrium where ROS is beneficial for tissue regeneration, though when their concentration goes past this point, adverse effects begin to rise and overshadow all advantageous characteristics [200]. Figure 3 summarizes the applications of ZnO-based nanostructures in tissue regeneration and healing.

### 5.4. ZnO Nanostructures for Drug Delivery

ZnO based nanoparticles which are the most significant metal oxide nanomaterials, are widely used in an increasing number of industrial products in various fields. Nano-ZnO composites lack toxicity and are easily absorbed by the body. This high bio-compatibility is in part explained by the fact that Zn is naturally present in all human tissues [201].

Multifunctional nanocarriers are under the microscope of the scientific community since they carry many benefits compared to traditional drugs. Nanomaterials can trigger cell and tissue-specific drug release through the application of an external stimulus [202], without the toxic adverse effects of traditional chemotherapeutics [24]. These nanostructures can deliver more than one drug simultaneously and they are easily degradable in the context of tissue microenvironment. The latest bibliographic data indicate that treatment with nanocarriers requires lower concentrations of the used drug as compared to individual compounds [24].

The design and development of various delivery systems is based on the different conditions (e.g., pH, temperature) that are expected to detect for targeted and controlled release of the drug. Temperature-controlled drug release has garnered significant interest due to its independence from alterations in certain chemical characteristics of the surrounding tissue microenvironment that may pose challenges in intracellular or in vivo settings [203]. Moreover, in pH-responsive systems, pH-sensitive linkers are typically used 2, and since blood and normal tissues have pH values that are significantly higher than those of tumors and inflammatory tissues, pH-responsive drug delivery methods provide exceptional benefits too [203].

One of the latest challenges the scientific community has confronted is the design of temperature and pH-sensitive nanoparticles. ZnO nanomaterials were first recommended as a type of pH-responsive drug carrier in 2010 [203] and were also suggested as nanocomposites with temperature-sensitive properties [202].

Numerous preceding scientific works have attempted the design of ZnO-based nanocomposites for drug delivery purposes. ZnO nanoparticles can be loaded with several drugs, including doxorubicin, paclitaxel, curcumin, and baicalin, as well as DNA fragments, for enhanced solubility, decreased toxicity as compared to individual compounds, and more efficient transport into cancer cells [201].

Firstly, Thomas et al. synthesized magnetic Zn-Fe_3_O_4_@SiO_2_ nanocomposites and found that under a magnetic field, internal heating induction occurred, leading to the release of their drug content [204].

In another study, the researchers designed multifunctional Fe_3_O_4_@ZnO:Er^3+^, Yb^3+^@β-CD nanocarriers with a core–shell structure. This structure makes possible the controlled drug release by magnetic targeting and microwave irradiation [202].

Furthermore, Zhao et al. synthesized ZnO@polymer core–shell nanoparticles encapsulated into novel capsule shells with 100 nm diameters to load isotretinoin (ISO). This drug-delivery system was pH-triggered and exhibited high effectiveness in killing cancer cells [205].

In a recent study, the scientific team designed a mesoporous TiO_2_@ZnO and TiO_2_@ZnO-GO nanocomposite which can be loaded with the chemotherapeutic drug of curcumin (CUR). The release of CUR was pH-dependent, and this nanostructure had beneficial effects on the elimination of colon cancer cells [206].

Additionally, Puvvada et al. created a ZnO nanocarrier (HZnO) which surface was conjugated with folic acid (FA) and allowed the successful targeting of the cells. This nanostructure was loaded with paclitaxel (PAC) and presented toxicity in MDA-MB-231 cells and shrank MDA-MB-231 xenograft tumors in mice. The cellular uptake of this nanostructure by breast cancer cells was folate receptor-mediated [207].

Moreover, a recent scientific work, presented albumin-grafted, polycaprolactone-coated, zinc oxide-loaded cloxacillin (APCL-CLOX-ZnO) nanoparticles, which were designed via a coaxial electrospray technique. This innovative coaxial nanostructure may serve as a promising drug delivery system for the treatment of bacterial infections, as well as for respiratory pathologies [208].

The design of nanoparticles that would successfully deliver doxorubicin (Dox) to the targeted cells has been a challenge for plentiful scientific works over the last years. More specifically, Zhuo et al. manufactured mesoporous silica nanoparticles (MSNs) that included Doxorubicin (Dox) inside their pores. The ZnO quantum dots (QDs) covered these pores and in pH = 5 the latter were released, so that DOX could be activated [209]. Hariharan et al. designed ZnO nanoparticles using a coprecipitation method and these structures were modified with a polyethylene glycol solution (PEG 600) (ZnO/PEG NPs). This ZnO/PEG nanosphere successfully imported Dox in a pH-related manner not only in HeLa cancer cells, but also in Gram-positive and negative bacteria. The latter was succeeded after photoactivation of these nanocomposites [210].

ZnO nanoparticles were also produced by Deng and Zhang using this chemical coprecipitation approach. These NPs were then employed to transport Dox creating a Dox-ZnO nanostructure. Dox-ZnO nanocomplexes proved to be a very effective drug delivery mechanism for the transfer of Dox into SMMC-7721 cells. Of note, Dox-ZnO nanocomplexes enhanced cell death in response to ultraviolet (UV) light due to their photocatalytic capabilities and their ability to synergistically induce caspase-dependent apoptosis [211].

The stealth effect significantly enhances the capabilities of nanomaterials for drug delivery in terms of improving pharmacokinetics, including blood circulation, biodistribution, and tissue targeting. After the analysis of Wen et al., it was proposed that structural holism can significantly improve the stealth effect, emphasizing the importance of considering the entire surface structure and geometry rather than relying solely on factors like maximizing repulsion force through polymer-based steric stabilization (e.g., PEGylation) or inhibiting immune attack through a bio-inspired component [212]. In addition, Cai et al. presented a pH-responsive nanocluster derived from ZnO quantum dots for targeted drug delivery to tumors, featuring individual ZnO QDs cross-linked with dicarboxyl-terminated PEG for stability and biocompatibility in physiological fluids. These nanoclusters efficiently encapsulated DOX and released it upon cellular uptake, inducing cytotoxicity in cancer cells post-dissolution, suggesting potential tumor- specific accumulation via the enhanced permeability and retention effect, while circulating the bloodstream [213].

**Figure 4:**
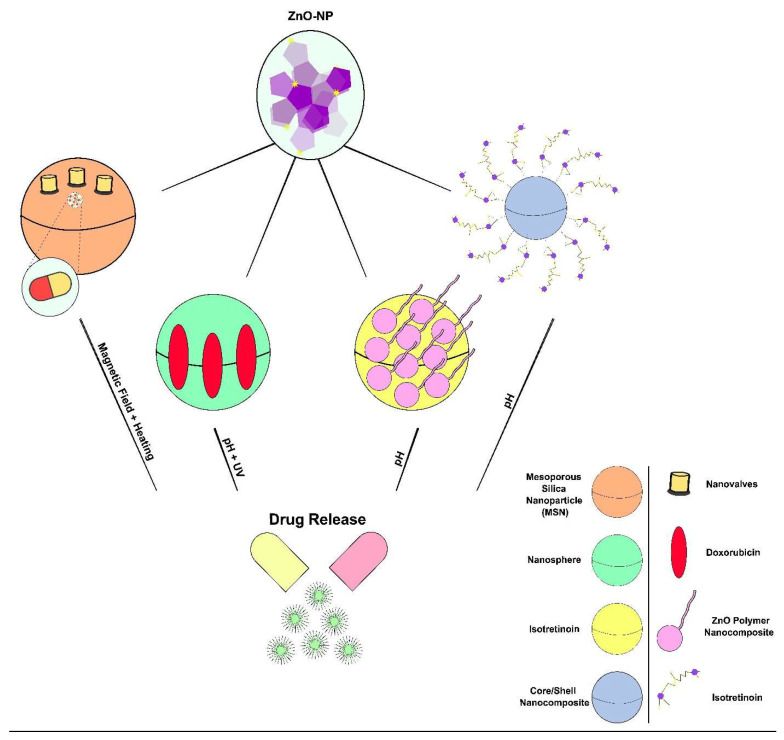
Different drug release mechanisms of ZnO functionalized nanocomposites.

Finally, Yang et al. synthesized red fluorescence ZnO nanoparticles with the chemical method of polyol in boiling trimethylene glycol (TREG) with zinc acetate. This structure was layered with polyglycerol (PG) and then was conjugated with arginine-glycine-aspartate (RGD). The nanocomposite was loaded with DOX and induced damage in U87MG cells [214]. Figure 4 summarizes the different drug release mechanisms of ZnO-based nanocomposites.

### 5.5. Applications of ZnO-Based Nanocomposites for Vaccines and Immunotherapy

To provoke an effective immune response, several steps are essential for inducing appropriate adaptive immunity against specific protein antigens. This involves the efficient delivery of the antigen into Antigen-Presenting cells (APCs), particularly dendritic cells and optimal processing and presentation to T cells. An effective vaccine system must navigate the intricate processes of antigen delivery to APCs, activating dendritic cells functionally and guide T cells towards specific differentiation. The growing number of nanotechnology-based vaccines, with diverse compositions, sizes, shapes, and surface properties, either approved or in human use, reflects advancements in this field [215,216].

In their research, Cho et al. created an iron oxide-zinc oxide-core shell nanoparticles that not only delivers carcinoembryonic antigen into dendritic cells but also serves as an imaging agent. The complex is swiftly taken by dendritic cells, detectable in vitro via confocal microscopy and in vivo through magnetic resonance imaging. Mice immunized with dendritic cells containing the nanoparticle-antigen complex exhibited heightened tumor antigen-specific T-cell responses, delayed tumor growth and improved survival rates compared to control one [217].

In 2017, a research team investigated the bioactivity of poly I:C (pIC) RNA bound to anticancer zinc oxide nanoparticles in the context of melanoma. The direct association of RNA with unfunctionalized ZnO nanoparticles was demonstrated by changes in size, zeta potential, and absorption/fluorescence spectra upon complexation. The RNA corona was visualized by transmission electron microscopy (TEM) for the first time. The complex exhibited enhanced cell death in human (A375) and mouse (B16F10) cell lines, suppressing tumor cell growth in a BALB/C-B16F10 mouse melanoma model. Ex vivo tumor analysis revealed significant molecular activity, including alterations in phosphoproteins and inflammation markers IL-6 and IFN-γ. High-throughput proteomics analysis identified zinc oxide and poly I:C-specific patterns, suggesting potential utility as an anticancer and immunotherapeutic strategy against melanoma [218].

Furthermore, in 2019, Sharma et al. showed that zinc oxide-based nanocomposites, particularly radially grown ZnO nanowires on poly-l-lactic acid (PLLA) microfibers, exhibit significant potential for vaccine development. This 3-dimensional structure demonstrates mild cellular toxicity while efficiently delivering tumor antigens into dendritic cells. These cells, acting as bridges between innate and adaptive immunity, are stimulated to express inflammatory cytokines and activation surface markers. The hybrid nanocomposites effectively induce tumor antigen-specific cellular immunity, resulting in substantial inhibition of tumor growth in vivo. Furthermore, the ZnO nanowires on PLLA fibers contribute to the systemic reduction of immune suppressive TReg cells and enhanced T cell infiltration into tumor tissues. This study highlights the promising biomedical applications of inorganic metal oxide-inert organic hybrid nanocomposites as an innovative vaccine platform [219].

## 6. Toxicity of ZnO Nanoparticles

Nano-ZnO particles exhibit toxicity across several organisms. They display a higher toxicity towards Gram-positive bacteria like *S. aureus*, in comparison with Gram-negative bacteria like *E. coli* and *P. aeruginosa*, owing to variations in their cell wall compositions [220]. Additionally, nano-ZnO particles impede the growth of algae [221], as evidenced by Brayner et al.’s study, where they restrained cyanobacteria’s photosynthetic capability [222]. Furthermore, nano-ZnO particles demonstrate toxicity in numerous higher plants like radish, lettuce, ryegrass, corn, wheat, and cucumber. This toxicity affects roots’ growth and seeds’ sprouting, ultimately leading to biomass decrease [221]. Likewise, nano-ZnO particles have been found to be toxic to lower vertebrates, such as aquatic crustaceans [223], *C. elegans* [224], and soil arthropods [225]. Exposure to nano-ZnO particles has been also observed to impact zebrafish’s development and embryogenesis [226].

ZnO nanoparticles’ toxicity on mammals has been observed in laboratory studies, as well as in living organisms. Within cells, nano-ZnO particles dissolve inside lysosomes [227], triggering processes such as cytotoxicity, oxidative stress, lipid peroxidation, enhanced intracellular Ca^2+^ levels, mitochondrial membrane’s potential alterations, lysosomal instability, release of inflammatory cytokines, DNA damage, leading ultimately to cell death [228]. When inhaled, these nanoparticles enter the lungs and then circulate through the bloodstream to other organs, exerting harmful impacts. Lung fluids’ acidic environment facilitates dissolution of particles, leading to inflammatory responses in the lungs [229]. Despite that the primary exposure route for nano-ZnO particles is through external (local) use, reports of skin inflammation are scarce [230]. However, intravenous infusion of nano-ZnO particles induces their distribution throughout the body, affecting organs such as the liver, spleen, lungs, heart, and kidneys. Interaction with these nanoparticles leads to histopathological damages, oxidative stress, tissues’ inflammation [231], as well as neurotoxicity in older mice [232].

Zinc oxide nanoparticles find extensive application in cosmetics, particularly in sunscreens. Numerous studies have highlighted the potential cytotoxic and genotoxic effects of nano-ZnO particles present in sunscreens, intended to provide shielding from UV irradiation [233]. Thus, a growing concern is underscored as the solutions meant for protection could potentially lead to adverse effects.

### 6.1. Neurotoxicity

ZnO nanoparticles are prevalent in paints, cosmetics, electronic devices, etc., posing a significant potential for exposure given their extensive incorporation into commercially available products. Despite their extensive usage, our understanding of the neurotoxic effects of nano-ZnO particles remains limited [234]. While studies have explored the interactions of nano-ZnO particles in vitro, the focus primarily revolves around neuronal cells. Research indicates that ZnO nanoparticles can penetrate PC-12 cells, leading to mitochondrial dysfunction and cell death [234]. Additionally, these nanoparticles induce toxicity that depends on the dose, as well as apoptosis in neuronal stem cells, through dissolution of the particles [235]. Moreover, exposure to ZnO NPs triggers cell cycle arrest and diminishes cell viability in RSC96 rat Schwann cells, with toxicity varying based on time, dose, and nanoparticle shape. Specifically, spheres exhibit heightened toxicity and prompt apoptosis within 12 h, while flower- and prism-shaped nanoparticles induce toxicity after extended exposure (48 h), attributed mainly to the anionic fraction of Zn rather than the nanoscale particulate fraction [236]. Furthermore, in human neuroblastoma cells (SH-SY5Y), nano-ZnO particles provoke oxidative stress, genotoxicity, alterations in the cells’ cycle, as well as apoptosis in a manner contingent upon both time and dosage. However, this research falls short in addressing cell’s internalization of particles [237].

Metal oxide nanoparticles can access the brain through breaching the blood–brain barrier or via an olfactory neuronal pathway. Research conducted by Kao et al., demonstrates the transfer of nano-ZnO particles across the brain through the olfactory neuronal pathway, evidenced by particles’ accumulation in rats’ olfactory bulbs following an exposure for 6 h [234]. Slight increases in brain’s Zn content were observed within the framework of a research upon recurrent oral administration of nano-ZnO particles [238]. In mice exhibiting depressive behavior, nano-ZnO particles reached the brain through both oral and inhalation exposure, disrupting spatial learning and memory by modifying synaptic plasticity [239]. Intravenous administration of nano-ZnO particles induced oxidative stress, impaired learning and memory, triggered inflammatory cytokine generation, and led to brain’s pathological lesions in an age-dependent manner [232]. Recurrent oral administration of nano-ZnO particles at a dosage of 600 mg/kg, resulted in oxidative stress and elevated inflammatory cytokines within the brain, with the toxicity exacerbated by concurrent oral administration of antioxidant molecules [240], albeit the dosage administered was considerably high compared to real-life exposure scenarios. In another study, rats injected intraperitoneally with nano-ZnO particles exhibited no signs of weight loss or behavioral alterations, but a statistically significant reduction in Fe and Ca levels within the brain tissue was observed [241]. The as-mentioned findings collectively suggest that exposure to nano-ZnO particles can lead to direct, as well as indirect detrimental effects on the brain.

### 6.2. Glial Cell Toxicity

Glial cells, pivotal in brain function, manage homeostasis, metabolism, and immune responses. Any deviation or reaction of these cells to injury can exacerbate neuronal harm [242]. Glial cells also maintain barrier and immune functions, positioning them as the brain’s first line of defense against nanoparticles. Thus, investigating nanoparticles’ interactions with glial cells is crucial.

The brain’s major glial cells are astrocytes and microglia. Microglia originate from hematopoietic stem cells, while astrocytes derive from neural stem cells [243]. Microglia, brain-resident immune cells, surveil for infections and injuries, entering the brain during primary development. They assist in synaptic remodeling by eliminating apoptotic neurons and secrete trophic factors crucial for neuronal well-being. Microglia may also modulate neuronal activity through receptors, clearing foreign bodies via ROS production [244]. However, excessive ROS can cause oxidative stress and damage neurons [245]. Exposure to nano-TiO_2_ particles triggered sudden oxidative bursts and mitochondrial dysfunction in microglia [246]. Microglial demise has been associated with neurodegenerative conditions like Alzheimer’s and schizophrenia [247]. Notably, research on nano-ZnO particles’ interaction with microglia remains lacking.

**Figure 5:**
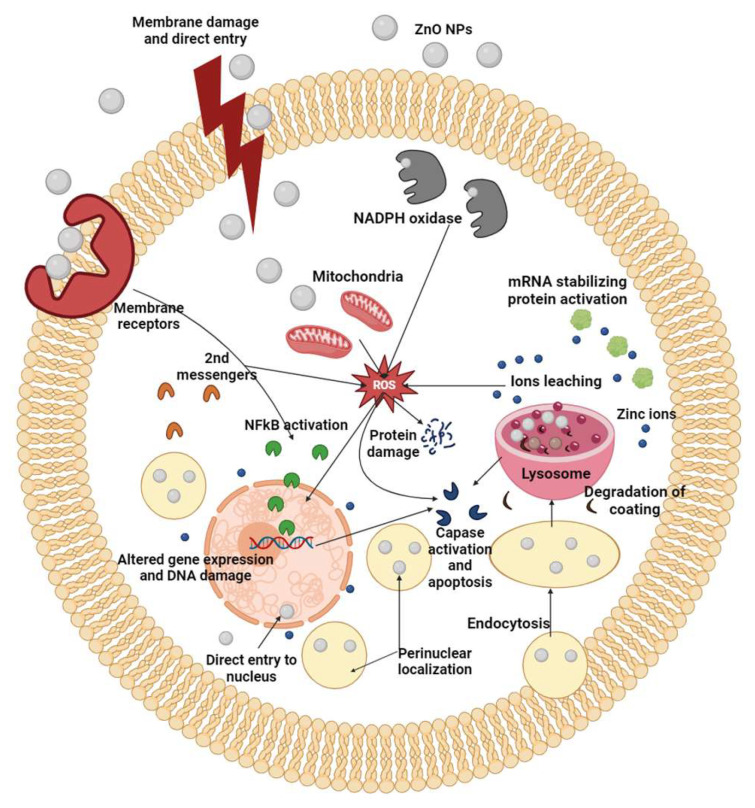
Schematic representation of the potential toxicity mechanism of ZnO nanoparticles (ZnO NPs) towards glial cells.

Astrocytes, the predominant glial cells within the brain, undertake various central nervous system functions, including axon guidance, synaptic support, blood–brain barrier maintenance, and metal balance regulation [248]. During brain injury, astrocytes aid in long-term recovery by expressing surface molecules and releasing trophic factors. Reactive astrogliosis, delineated by increased expression of glial fibrillary acidic protein (GFAP) [249], signifies astrocyte activation in response to stress and brain trauma. Research indicates that nanoparticles exert toxicity on astrocytes. In rats, repeated intraperitoneal exposure to a concentration equal to 1 mg/kg of Al_2_O_3_ nanoparticles led to GFAP upregulation, with significant effects compared to bulk Al_2_O_3_ [250]. Conversely, titanium dioxide nanoparticles induce astrocyte senescence and caspase-mediated apoptosis [251]. Dysregulation of metal balance and astrocyte apoptosis have implications for neurodegenerative diseases [252]. Despite their pivotal role in brain zinc homeostasis, studies on the interaction between nano-ZnO particles and glial cells remain limited compared to neurons. Preliminary investigations suggest the toxicity of nano-ZnO particles to glioma cells [252], highlighting the need for further research on their interaction with glial cells (Figure 5).

### 6.3. Reactive Oxygen Species (ROS) Generation

Nanoparticles toxicity is contingent upon the interaction between nanoparticles and biological systems, dictated by their physicochemical attributes. Given their nano-size, enhanced surface area to volume ratio, as well as quantum confinement, nanoparticles interact with biological systems in unforeseeable ways. For example, while Au is typically inert, nano-Au particles exhibit enhanced reactivity [253]. Their small size enables them to traverse the body and reach protected regions like the cell nucleus, blood–brain barrier, and placenta [254]. Zinc oxide nanoparticles penetrate cells via direct diffusion or endocytosis, triggering ROS production and oxidative stress-induced damage upon interaction with biological systems. The extensive surface area of nano-ZnO particles exposes numerous reactive sites, fostering high surface reactivity and consequent damage, including protein impairment, DNA damage, lipid peroxidation, inflammation, as well as organelle dysfunction. While cells own intrinsic antioxidant mechanisms for counteracting ROS produced during normal metabolism, the presence of nano-ZnO particles may overwhelm these mechanisms, leading to pro-oxidant behavior and oxidative stress induction through ROS generation or inhibition of antioxidant molecules. Various mechanisms contribute to ROS production: (a) the direct engagement of nano-ZnO particles with acidic compartments like lysosomes leads to the release of toxic ions, subsequently initiating the generation of ROS through chemical reactions, (b) nano-ZnO particles disrupt the mitochondrial electron (e^−^) transport chain and oxidative phosphorylation processes, culminating in ROS’ production, (c) nano-ZnO particles interact with redox-active enzymes like NADPH oxidase, inducing oxidative stress and (d) nano-ZnO particles may bind with cellular receptors, initiating downstream signaling pathways like the activation of Nuclear Factor-κB (NF-κB).

Additional potential consequences of ZnO nanoparticles interaction involve the dissolution of particles and subsequent release of toxic ions, disrupting biological system’s normal functions [255]. The dissolution of nano-ZnO particles within cellular organelles disturbs metal balance, while free ions may impede enzyme processes. Nanoparticles’ localization around the nucleus interferes with cells’ transcription and translation processes. A variety of mRNA-stabilizing enzymes possess metal-responsive regions, which are triggered when metal ions are present, thus disrupting the transcriptome. Nanoparticles like gold can modify cells’ gene expression profile through directly interacting with DNA and stimulating oxidative stress. Furthermore, nanoparticles surficially adsorb proteins, developing a protein corona. The as-absorbed proteins might encounter structural alterations, rendering them unrecognizable as native proteins by the immune system, thus provoking immune responses.

Numerous studies have addressed the toxicity related with nano-ZnO particles, primarily attributed to particle dissolution and the consequent ROS’ production. The supplementation of antioxidants has been shown to alleviate the toxic effects induced by nano-ZnO particles [256]. Moreover, doping with Fe enhances nano-ZnO particles’ stability, leading to reduced dissolution and diminished toxicity observed in zebrafish, as well as rodent lungs [257]. Nano-ZnO particles’ dissolution in an aqueous solution is generally described by the following equations (Equations (3)–(5)) [258]:

(3) $${\mathrm{ZnO}}_{\left(s\right)}+H_{2}O_{\left(l\right)}{↔\mathrm{Zn}{\left(\mathrm{OH}\right)}_{2}}_{\left(s\right)}$$

(4) $$\mathrm{Zn}{\left(\mathrm{OH}\right)}_{2}↔\mathrm{Zn}{\left(\mathrm{OH}\right)}_{\left(\mathrm{aq}\right)}^{+}+{\mathrm{OH}}_{\left(\mathrm{aq}\right)}^{-}$$

(5) $$\mathrm{Zn}{\left(\mathrm{OH}\right)}_{\left(\mathrm{aq}\right)}^{+}↔{\mathrm{Zn}}_{\left(\mathrm{aq}\right)}^{2+}+{\mathrm{OH}}_{\left(\mathrm{aq}\right)}^{-}$$

### 6.4. Oxidative Stress

Reactivity, as well as reactive oxygen species production can originate from the triggering of electron (e^−^)/hole (h^+^) pairs or crystal defects as oxygen vacancies, as well as enhanced interstitial Zn within nanoparticles [259]. The as-mentioned nanoparticles directly engage with biomolecules like lipid membranes, inducing the generation of superoxide (•O_2_^−^) radicals. Both nano-ZnO particles and the Zn^2+^ ions emitted through dissolution, interact with cellular organelles, like mitochondria and the endoplasmic reticulum. Exposure to these nanoparticles leads to mitochondrial membrane’s potential loss and the activation of mitogen-activated protein kinase (MAPK) pathways, ultimately triggering apoptosis [260]. Additionally, Zn^2+^ interference with the e- transport chain exacerbates the generation of ROS. The •O_2_^−^ radicals produced by nano-ZnO particles possess high potency in damaging biomolecules, causing DNA damage and increased expression of death receptor genes [261]. The as-mentioned ROS production interrupts oxidants-antioxidants balance within cells, resulting in oxidative stress.

Within cells, nano-ZnO particles dissolve, releasing Zn^2+^ ions. Transition metal ions, such as Zn and Fe, serve as essential co-factors for numerous proteins, playing critical roles in their enzymatic functions. Zinc, in particular, acts as a cofactor for approximately 300 enzymes within the body [262]. These Zn ions possess the capacity to produce chelates with coordinating molecules within enzymes, consequently modifying protein’s catalytic activity. Such interactions often lead to proteinic structural and functional alterations [263]. For example, Zn^2+^ may displace Mg^2+^ from active sites, thus resulting in protein deactivation [264]. Additionally, Zn^2+^ interacts with metal-responsive regions of mRNA-stabilizing proteins, leading to disruption of cellular mRNA [263].

The dissolution of particles within cells disrupts zinc balance and increases the production of reactive oxygen species (ROS) [265]. This heightened zinc concentration subsequently induces oxidative stress, disrupts protein folding, damages mitochondria, deactivates lysosomes, inhibits enzymes, induces genotoxicity, and ultimately leads to cell death [266]. Imbalances in zinc homeostasis also affect the functions of Zn-containing enzymes [267]. Moreover, studies have indicated that nano-ZnO particles can reduce the activity of Ca ATPase within the plasma membrane, resulting in an elevation of intracellular Ca levels. This elevated calcium concentration disrupts calcium balance, initiating mitochondrial dysfunction and ultimately leading to cell death [268].

However, surface modification with hydrophilic polymers will decrease the toxicity, as it is indicated in the investigation of a scientific team that examined ZnO nanoparticles disposition within human immune cells, revealing that PEGylation reduces toxicity by limiting cellular uptake, while surface properties primarily regulate nanoparticle uptake rather than intracellular or extracellular Zn dissolution levels [269].

### 6.5. Toxicity-Involved Principal Pathways

**Figure 6:**
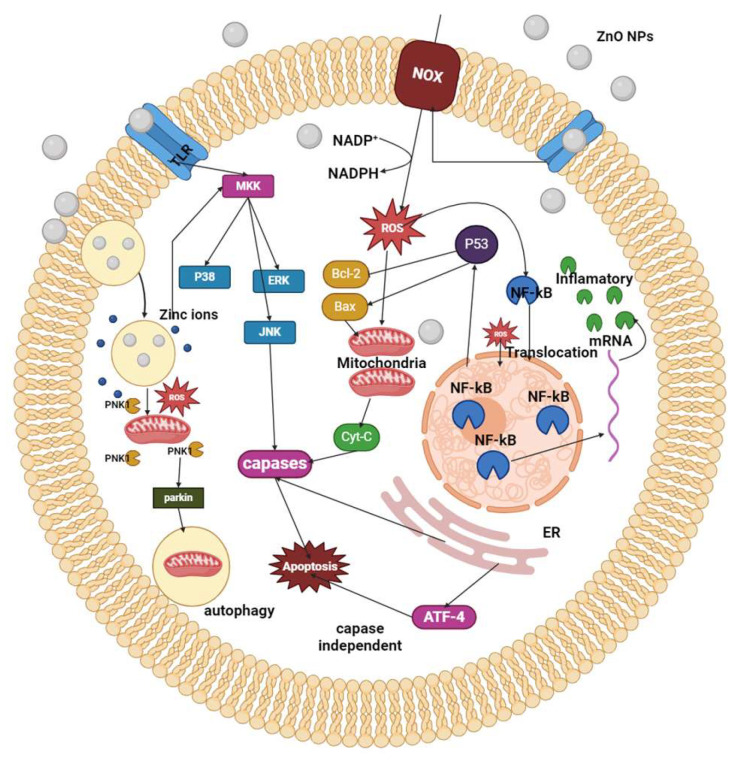
Illustration of the potential cell signaling pathways that are involved in the ZnO nanoparticles (ZnO NPs)-induced toxicity.

Nano-ZnO particles induce toxicity through various cellular signaling pathways, which are contingent upon the cell type and the degree of toxicity. The primary pathways involved in the interaction between nano-ZnO particles and biological systems are illustrated in Figure 6.

One such pathway is the redox pathway, which entails the production of free radicals by NADPH oxidase (NOX). NOX enzymes, located on cell membranes, catalyze the production of •O_2_^−^ anions through the reaction entailing NADPH and molecular oxygen. The contribution of the as-mentioned signaling mechanism in nano-ZnO particles’ toxicity is crucial, as it consistently leads to an enhancement in ROS production. Several research projects have implied that exposure to nano-ZnO particles triggers NOX-dependent ROS production within macrophages [270]. Nevertheless, the relationship between NOX and apoptosis in macrophages remains unestablished in some research. Furthermore, it has been noted that, in addition to mitochondria, NOX has a substantial impact on nano-ZnO particles-induced ROS generation [262]. Although a plethora of studies do not explicitly refer to NOX in the context of nano-ZnO particles’ toxicity, the cellular redox state and the involvement of surficial cell receptors that interact with NOX are frequently discussed [252].

P53, a pivotal tumor suppressor gene governing the cell cycle, plays a crucial role in responding to DNA damage by initiating repair mechanisms or apoptotic pathways founded on damage’s severity. Research has demonstrated that exposure to nano-ZnO particles triggers activation of P53, leading to apoptosis in skin fibroblast (BJ cells) and alveolar epithelial cells (A549 cells) [271].

MAPKs, a group of serine/threonine kinases essential for fundamental cellular functions like growth, proliferation, alteration, and programmed cell death, contribute significantly in mediating the toxic impacts of nano-ZnO particles. Researches have illustrated that nano-ZnO particles provoke activation of various MAPK pathways, including ERK, p38, and SAP/JNK kinases, particularly in human lung carcinoma cells. Notably, the apoptotic pathway SAP/JNK was predominantly prompted by Zn^2+^ ions’ release from nano-ZnO particles within these cells [272]. Similarly, primary astrocytes exposed to nano-ZnO particles exhibited activation of all aforementioned MAPK pathways, with apoptosis primarily mediated through the JNK signaling pathway. In this context, inhibition of JNK notably mitigated the toxic effects. Additionally, in macrophages, ZnO nanoparticles-induced MAPK activation through TLR6 receptors [273], illustrating that the mechanism underlying nano-ZnO particles-triggered MAPK stimulation is dependent from the cells’ type.

Under specific stress conditions, the environment within the endoplasmic reticulum can undergo changes, impacting the functionality of molecular chaperone proteins and disrupting the normal protein-folding process [274]. Consequently, a signaling cascade referred to as UPR is triggered to address endoplasmic reticulum stress and restore optimal conditions for protein folding. Studies indicate that exposure to nano-ZnO particles triggers endoplasmic reticulum stress response in laboratory experiments, as well as in living organisms, leading to hepatotoxicity in mice [275]. Researchers have identified endoplasmic reticulum stress as an early toxicity indicator of nano-ZnO particles [276], noting that even non-cytotoxic levels of these nanoparticles can induce endoplasmic reticulum stress. Moreover, analysis of gene expression patterns in cells exposed to nano-ZnO particles reveals changes in genes associated with chaperonin and protein-folding functions, regardless cells’ type [277].

PINK1, a protein kinase found within mitochondria, serves to shield cells from mitochondrial dysfunction induced by stress [278]. Researchers have shown that the PINK1/parkin pathway is involved in nano-ZnO particles-induced toxicity [279]. The obtained findings revealed that exposure to ZnO nanoparticles increases the expression of PINK1 protein and mitochondrial parkin levels. The as-acquired data suggest that the PINK1/parkin pathway serves a protective role in BV-2 cells exposed to nano-ZnO particles, as evidenced by lower survival rates observed in PINK1 knockout cells when nano-ZnO particles are present. PINK1 typically undergoes degradation after being introduced into healthy mitochondria’s inner membrane. However, in depolarized mitochondria, accumulation of PINK1 takes place on the outer membrane and recruits parkin to initiate autophagy.

Finally, nuclear factor-κB (NF-κB) comprises a set of transcription factors responsible for regulating genes linked to various processes such as inflammation, response to stress, cell viability, adaptive immunity, as well as the development of lymphocytes. These factors play crucial roles in cellular reactions triggered by stimuli like mechanical stress, the generation of free radicals, and inflammation [280]. Detecting NF-κB activation typically involves assessing gene expression levels and observing the movement of cytoplasmic NF-κB into the nucleus. Nano-ZnO particles primarily induce toxicity through the generation of reactive oxygen species. Recent findings by Liang et al. suggest a potential association between nano-ZnO particles and lung inflammation via ROS generation and the triggering of NF-κB [281]. Moreover, nano-ZnO particles have been observed to prompt NF-κB translocation in human epithelial tubular (HK-2) cells [282]. Additionally, a significant study has revealed that nano-ZnO particles can penetrate ovarian tissue and impede embryonic growth during the oocyte stage by activating NF-κB [283].

## 7. Discussion-Conclusions

In conclusion, the rapid rise of nanotechnology has shifted focus from single materials to nanohybrids, encompassing various nanostructures, such as nanorods, nanowires, hollow spheres and nanoflowers. Nanostructured ZnO materials with unique crystalline and surface structures, along with notable properties like high conductivity, gas sensitivity, biocompatibility and antibacterial activity, hold significant promise in biomedicine. Recent reports emphasize the importance of comprehensively understanding the mechanisms of ZnO nanomaterials.

Key physiochemical and optical characteristics of nanohybrid materials, including a large surface area and a high isoelectric point, contribute to high adsorption efficiency, non-toxicity, biocompatibility, mechanical and chemical stability, catalytic efficiency and electrical conductivity. These properties are advantageous for developing efficient and high-performance biosensor devices. ZnO based biosensors are poised to revolutionize biomedical diagnostics.

The versatility of ZnO nanostructures is evident in their integration into biosensors for both non-enzymatic and enzymatic applications. Modifying ZnO surfaces with nanostructured transition metal oxides or creating ZnO-metal oxide nanoparticles enhances catalytic capabilities, crucial for improving biosensor performance and ensuring efficient target analyte detection. In glucose biosensors, ZnO-based materials offer exceptional sensitivity, stability, and rapid response, particularly beneficial for monitoring glucose levels in individuals with diabetes. Non-enzymatic glucose sensors use techniques like electrospinning and metal oxide incorporation to enhance electrocatalytic activity. The selection and design of nanomaterials play a crucial role in rapid and accurate biomolecule detection. Advances in material synthesis approaches, enzyme and protein engineering and conjugation strategies continue to yield novel nano-engineered segments with improved functionality. Emerging manufacturing technologies, such as nanoscale-oriented 3D or 4D printing of multifunctional nanostructures, are expected to bring new dimensions to sensor design. Research in advanced biosensing with biofunctionalized multifunctional nanomaterials and the development of cost-effective biochip designs employing nanoscale sensing materials can pave the way for economical lab on a chip replacement for real time biomolecule sensing.

Despite notable advancements, challenges persist, such as reproducible mass production of ZnO nanocomposites, understanding nano-bio interfaces and addressing potential toxicity during in vivo applications.

Moreover, conventional methods for producing ZnO QDs exhibit drawbacks. Including low quantum yield and broad photoluminescence bands. The instability of ZnO QDs in aqueous solution can be addressed through surface modification with specific ligands. Recent advancements in the field underscore the potential of modified ZnO nanomaterials for diverse biomedical applications from cancer imaging to cell imaging. Despite these achievements, ongoing research is essential to overcome challenges and further optimize the performance of ZnO-based QDs for various bioimaging applications.

Furthermore, these functionalized nanoparticles exhibit remarkable potential as antimicrobial agents against a wide range of pathogens, demonstrating bactericidal and fungicidal properties. The mechanisms through ZnO exerts its antimicrobial effects include targeting bacterial membranes, enhancing ROS production and causing damage by through Zn^2+^ release. The effectiveness of these properties is dependent on nanoparticles’ concentration and size, with smaller particles proving more efficient. In the context of wound healing and bone implants, ZnO-based nanostructures, particularly those with piezoelectric properties, show promise for electrical cell stimulation. However, challenges persist, such as finding the optimal concentration of ZnO to balance its beneficial effects on angiogenesis while avoiding adverse effects associated with elevated ROS concentrations.

Regarding drug delivery systems, ZnO functionalized nanoparticles exhibit wide applications, owing to their high biocompatibility rooted in zinc’s natural presence in human tissues. These nanostructures offer controlled drug release, responding to stimuli like temperature and pH, showcasing lower drug concentrations, reduced toxicity and improved solubility compared to traditional compounds. The versatility of ZnO nanocarriers is highlighted in various formulations, while the refinement of these nanocarriers hold promise for advancing drug delivery technology.

Lastly, the pursuit of an effective immune response to specific protein antigens involves precise steps in inducing adaptive immunity, emphasizing optimal antigen delivery. The field of nanotechnology-based vaccines, marked by diverse compositions and properties, signifies substantial progress and shapes a future of vaccine development for enhanced immune responses to specific antigens.

## Figures and Tables

**Table 1:** ZnO nanostructures utilized for biosensing applications.

Nanocomposite	Sensor	Reference
ZnO NRs/ITO	Non enzymatic	[167]
ZnO-CuO/FTO	Non enzymatic	[168]
RGO/ZnO film	Non enzymatic	[169]
ZnO-CuO nanofibers	Glucose	[172]
Cu/CuO/ZnO	Glucose	[173]
ZnO/Fe_2_O_3_/FTO	Glucose	[174]
NiO QDs/ZnO	Glucose	[175]
ZnO/chitosan	Enzymatic	[176]
ZnO/Ag	Enzymatic	[177]

## Data Availability

Not applicable.
